# Supplementary material for: Tuberculosis alters immune-metabolic pathways resulting in perturbed IL-1 responses
Source: Front Immunol. 2022 Dec 14;13:897193. doi: 10.3389/fimmu.2022.897193 (PMC9795069; doi:10.3389/fimmu.2022.897193)
Supplement: Supplementary file 1 [file Table_1.docx]

| Plus genes |
| --- |
| ACTA2 |
| ALDH1A1 |
| ANKRD22 |
| APOL1 |
| APOL6 |
| BATF2 |
| KLF2 |
| CALML4 |
| CASP4 |
| CREB5 |
| CYB561 |
| DEFA1 |
| DUSP3 |
| ETV7 |
| GAS6 |
| GBP2 |
| GBP4 |
| GBP6 |
| HPSE |
| KCNJ15 |
| KREMEN1 |
| LACTB |
| LHFPL2 |
| LOC389386 |
| FER1L3 |
| SCARF1 |
| SEPT4 |
| SMARCD3 |
| TRAFD1 |
| VAMP5 |

**Supplementary Table S1**. Tuberculosis-related genes added to the Nanostring human immunology v2 panel.

| **Null (20)** | **BCG (28)** | **IL1b (15)** | **TB Ag (71)** | |
| --- | --- | --- | --- | --- |
| BAX | ADA | C1S | ACTA2 | IL27 |
| CD46 | ALDH1A1 | CR1 | ALAS1 | IL7 |
| CD86 | BATF | CYB561 | ANKRD22 | IRF1 |
| CLEC7A | C7 | DEFB103B | APOL1 | IRF8 |
| CMKLR1 | C8A | DEFB4A | APOL6 | JAK2 |
| CXCL1 | CCL22 | GZMA | BATF2 | KCNJ2 |
| HAVCR2 | CD22 | HLA.DOB | CALML4 | KLRG1 |
| IFNAR2 | CD36 | ICAM2 | CCL24 | LHFPL2 |
| IL10RA | CD99 | KLRK1 | CD274 | LILRB4 |
| IL12RB1 | CLU | NCR1 | CD276 | LOC389386 |
| IL1RL2 | CSF2 | NFKB1 | CD44 | LTB4R2 |
| KIT | CTNNB1 | NOD1 | CD45R0 | MUC1 |
| LILRA2 | CTSG | NT5E | CD74 | PDCD1LG2 |
| MME | IL10 | TNFRSF10C | CD83 | PECAM1 |
| PTGS2 | IL22 | TNFSF12 | CISH | PML |
| RPL19 | IL22RA2 |  | CLEC4A | PRDM1 |
| S100A8 | IL6 |  | CSF2RB | PSMB10 |
| STAT4 | IRF4 |  | CXCL2 | PTAFR |
| STAT6 | JAK1 |  | ETV7 | PTPN22 |
| TYK2 | LAIR1 |  | FAS | PTPRC |
|  | NCF4 |  | FCGR1A.B | SMAD3 |
|  | PRF1 |  | FER1L3 | SOCS1 |
|  | PSMB5 |  | FN1 | SOCS3 |
|  | PSMB7 |  | FYN | STAT1 |
|  | PSMC2 |  | GBP1 | STAT2 |
|  | PSMD7 |  | GBP4 | STAT5A |
|  | TRAF2 |  | GFI1 | TAP2 |
|  | sCTLA4 |  | GPR183 | TGFBI |
|  |  |  | HLA.C | TGFBR2 |
|  |  |  | HLA.DMA | TLR8 |
|  |  |  | HLA.DPB1 | TNFAIP6 |
|  |  |  | HLA.DRA | TNFSF10 |
|  |  |  | IFI16 | TNFSF13B |
|  |  |  | IFITM1 | TRAFD1 |
|  |  |  | IFNB1 | VAMP5 |
|  |  |  | IL21 |  |

**Supplementary Table S2**. List of differentially expressed genes between LTBI and TB -only after specific stimulation (t-test with a cut-off value of q<0.001).

|  | TB Ag | BCG |  |
| --- | --- | --- | --- |
| Different | 62 | 40 |  |
| No different | 560 | 582 | q=0.03 (*) |

|  | TB Ag | IL-1b |  |
| --- | --- | --- | --- |
| Different | 62 | 16 |  |
| No different | 560 | 606 | q=0.0001 (****) |

|  | BCG | IL-1b |  |
| --- | --- | --- | --- |
| Different | 40 | 16 |  |
| No different | 582 | 606 | q=0.002 (**) |

**Supplementary Table S3**. Contingency test (Fischer’s exact test) for the differentially expressed genes upon immune stimulation (BCG, IL-1β and TB Ag).

| q-value | | | | |
| --- | --- | --- | --- | --- |
| Transcript | Null | BCG | IL-1b | TB Ag |
| CLL7 | 0.0001 | 0.6145 | 0.4439 | 0.1676 |
| CCL5 | 0.8021 | 0.0006 | 0.6393 | 0.922 |
| NFKB1 | 0.001 | 0.1019 | 2.95 x 10^-4^ | 0.0026 |
| IDO1 | 0.0724 | 0.9277 | 0.443 | 2.24 x 10^-5^ |

**Supplementary Table S4**. Examples of stimuli-specific induced differences between LTBI and TB with q-value specified for each condition.

| \| **BIOCHEMICAL** \| **Pathway** \| **TB** \| **LTBI** \| \| --- \| --- \| --- \| --- \| \| 5alpha-pregnan-3beta,20alpha-diol monosulfate (2) \| Steroid \| 0.55 \| -0.06 \| \| 5alpha-pregnan-3beta,20beta-diol monosulfate (1) \| Steroid \| 0.55 \| -0.05 \| \| 5alpha-pregnan-3beta,20alpha-diol disulfate \| Steroid \| 0.55 \| -0.08 \| \| pregnanediol-3-glucuronide \| Steroid \| 0.51 \| -0.04 \| \| sphingomyelin (d18:1/20:2, d18:2/20:1, d16:1/22:2)* \| Sphingolipid Metabolism \| 0.43 \| -0.09 \| \| 5alpha-pregnan-3(alpha or beta),20beta-diol disulfate \| Steroid \| 0.40 \| 0.11 \| \| 4-ethylphenylsulfate \| Benzoate Metabolism \| 0.39 \| -0.02 \| \| stearoyl sphingomyelin (d18:1/18:0) \| Sphingolipid Metabolism \| 0.37 \| 0.26 \| \| 1-palmitoleoylglycerol (16:1)* \| Monoacylglycerol \| 0.36 \| -0.21 \| \| pyrraline \| Food Component/Plant \| 0.36 \| -0.02 \| \| tryptophan betaine \| Tryptophan Metabolism \| 0.35 \| -0.33 \| \| sphingomyelin (d18:1/22:2, d18:2/22:1, d16:1/24:2)* \| Sphingolipid Metabolism \| 0.34 \| -0.09 \| \| 3-hydroxybutyrylcarnitine (2) \| Fatty Acid Metabolism(Acyl Carnitine) \| 0.33 \| 0.20 \| \| 1-eicosapentaenoylglycerol (20:5)* \| Monoacylglycerol \| 0.32 \| 0.05 \| \| laurate (12:0) \| Medium Chain Fatty Acid \| 0.32 \| 0.12 \| \| N-delta-acetylornithine \| Urea cycle; Arginine and Proline Metabolism \| 0.31 \| -0.06 \| \| cystine \| Methionine, Cysteine, SAM and Taurine Metabolism \| 0.30 \| -0.10 \| \| indole-3-carboxylic acid \| Tryptophan Metabolism \| 0.29 \| 0.14 \| \| sphingomyelin (d18:1/18:1, d18:2/18:0) \| Sphingolipid Metabolism \| 0.29 \| 0.25 \| \| linoleoyl-docosahexaenoyl-glycerol (18:2/22:6) [1]* \| Diacylglycerol \| 0.28 \| 0.21 \| \| 1-linolenoylglycerol (18:3) \| Monoacylglycerol \| 0.28 \| -0.07 \| \| 1-arachidonylglycerol (20:4) \| Monoacylglycerol \| 0.27 \| -0.35 \| \| 1-docosahexaenoylglycerol (22:6) \| Monoacylglycerol \| 0.27 \| -0.11 \| \| betonicine \| Food Component/Plant \| 0.26 \| -0.08 \| \| sphingomyelin (d18:0/18:0, d19:0/17:0)* \| Sphingolipid Metabolism \| 0.26 \| 0.48 \| \| N-methylproline \| Urea cycle; Arginine and Proline Metabolism \| 0.24 \| -0.07 \| \| 4-methylcatechol sulfate \| Benzoate Metabolism \| 0.24 \| 0.03 \| \| isobutyrylcarnitine (C4) \| Leucine, Isoleucine and Valine Metabolism \| 0.24 \| 0.16 \| \| 1-arachidonoyl-GPA (20:4) \| Lysolipid \| 0.22 \| -0.27 \| \| p-cresol sulfate \| Phenylalanine and Tyrosine Metabolism \| 0.21 \| -0.32 \| \| gamma-glutamylglutamate \| Gamma-glutamyl Amino Acid \| 0.20 \| -0.30 \| \| 2-arachidonoylglycerol (20:4) \| Monoacylglycerol \| 0.19 \| -0.20 \| \| 1-palmitoyl-GPA (16:0) \| Lysolipid \| 0.18 \| -0.27 \| \| oleoyl-arachidonoyl-glycerol (18:1/20:4) [1]* \| Diacylglycerol \| 0.18 \| 0.30 \| \| stachydrine \| Food Component/Plant \| 0.17 \| -0.06 \| \| 1-palmitoyl-2-oleoyl-GPI (16:0/18:1)* \| Phospholipid Metabolism \| 0.17 \| 0.05 \| \| 2-palmitoleoyl-GPC (16:1)* \| Lysolipid \| 0.17 \| -0.02 \| \| tetradecanedioate \| Fatty Acid, Dicarboxylate \| 0.17 \| 0.33 \| \| oleoyl-arachidonoyl-glycerol (18:1/20:4) [2]* \| Diacylglycerol \| 0.17 \| 0.39 \| \| 1-palmitoyl-GPG (16:0)* \| Lysolipid \| 0.16 \| 0.15 \| \| linoleoyl-arachidonoyl-glycerol (18:2/20:4) [1]* \| Diacylglycerol \| 0.16 \| 0.01 \| \| methyl glucopyranoside (alpha + beta) \| Food Component/Plant \| 0.16 \| 0.21 \| \| linoleoyl-docosahexaenoyl-glycerol (18:2/22:6) [2]* \| Diacylglycerol \| 0.16 \| 0.23 \| \| gluconate \| Food Component/Plant \| 0.16 \| 0.11 \| \| 1-palmitoyl-2-linoleoyl-GPI (16:0/18:2) \| Phospholipid Metabolism \| 0.16 \| 0.18 \| \| glycocholenate sulfate* \| Secondary Bile Acid Metabolism \| 0.16 \| 0.20 \| \| sphingomyelin (d18:2/23:1)* \| Sphingolipid Metabolism \| 0.16 \| 0.24 \| \| undecanoate (11:0) \| Medium Chain Fatty Acid \| 0.15 \| -0.01 \| \| sphingomyelin (d18:2/24:2)* \| Sphingolipid Metabolism \| 0.15 \| -0.20 \| \| indolepropionate \| Tryptophan Metabolism \| 0.15 \| -0.04 \| \| 2-palmitoyl-GPC (16:0)* \| Lysolipid \| 0.15 \| -0.21 \| \| palmitoleoyl-linoleoyl-glycerol (16:1/18:2) [1]* \| Diacylglycerol \| 0.14 \| 0.18 \| \| sphingomyelin (d18:1/17:0, d17:1/18:0, d19:1/16:0) \| Sphingolipid Metabolism \| 0.14 \| 0.38 \| \| benzoate \| Benzoate Metabolism \| 0.14 \| 0.21 \| \| sphingomyelin (d18:2/18:1)* \| Sphingolipid Metabolism \| 0.14 \| 0.21 \| \| 1-myristoylglycerol (14:0) \| Monoacylglycerol \| 0.13 \| -0.13 \| \| 3-phenylpropionate (hydrocinnamate) \| Phenylalanine and Tyrosine Metabolism \| 0.13 \| -0.15 \| \| sebacate (decanedioate) \| Fatty Acid, Dicarboxylate \| 0.12 \| 0.43 \| \| isovalerylcarnitine (C5) \| Leucine, Isoleucine and Valine Metabolism \| 0.12 \| -0.22 \| \| choline \| Phospholipid Metabolism \| 0.12 \| 0.31 \| \| 1-palmitoyl-GPC (16:0) \| Lysolipid \| 0.11 \| 0.17 \| \| gamma-glutamylglutamine \| Gamma-glutamyl Amino Acid \| 0.11 \| -0.18 \| \| 1-oleoyl-GPA (18:1) \| Lysolipid \| 0.11 \| -0.24 \| \| 4-allylphenol sulfate \| Food Component/Plant \| 0.11 \| -0.26 \| \| 1-palmitoleoyl-GPC (16:1)* \| Lysolipid \| 0.10 \| 0.03 \| \| gamma-glutamylalanine \| Gamma-glutamyl Amino Acid \| 0.10 \| -0.31 \| \| undecanedioate \| Fatty Acid, Dicarboxylate \| 0.10 \| 0.10 \| \| docosapentaenoate (n3 DPA; 22:5n3) \| Polyunsaturated Fatty Acid (n3 and n6) \| 0.10 \| 0.07 \| \| 2-oxoarginine* \| Urea cycle; Arginine and Proline Metabolism \| 0.10 \| -0.16 \| \| isocitrate \| TCA Cycle \| 0.10 \| 0.05 \| \| 1-arachidonoyl-GPC (20:4n6)* \| Lysolipid \| 0.10 \| -0.19 \| \| pentadecanoate (15:0) \| Long Chain Fatty Acid \| 0.10 \| 0.19 \| \| 3-hydroxyhexanoate \| Fatty Acid, Monohydroxy \| 0.10 \| 0.44 \| \| palmitoyl-linolenoyl-glycerol (16:0/18:3) [2]* \| Diacylglycerol \| 0.10 \| 0.32 \| \| 1-palmitoylglycerol (16:0) \| Monoacylglycerol \| 0.10 \| -0.32 \| \| gentisate \| Phenylalanine and Tyrosine Metabolism \| 0.09 \| 0.11 \| \| diacylglycerol (16:1/18:2 [2], 16:0/18:3 [1])* \| Diacylglycerol \| 0.09 \| 0.33 \| \| myristoyl dihydrosphingomyelin (d18:0/14:0)* \| Sphingolipid Metabolism \| 0.09 \| 0.23 \| \| diacylglycerol (12:0/18:1, 14:0/16:1, 16:0/14:1) [2]* \| Diacylglycerol \| 0.09 \| 0.32 \| \| 1-palmitoyl-GPI (16:0) \| Lysolipid \| 0.08 \| -0.10 \| \| kynurenine \| Tryptophan Metabolism \| 0.08 \| -0.24 \| \| palmitoyl sphingomyelin (d18:1/16:0) \| Sphingolipid Metabolism \| 0.08 \| 0.30 \| \| 3-hydroxy-2-ethylpropionate \| Leucine, Isoleucine and Valine Metabolism \| 0.08 \| -0.25 \| \| 1-palmitoyl-2-palmitoleoyl-GPC (16:0/16:1)* \| Phospholipid Metabolism \| 0.08 \| 0.25 \| \| 4-hydroxyhippurate \| Benzoate Metabolism \| 0.07 \| 0.16 \| \| 1-palmitoyl-2-arachidonoyl-GPE (16:0/20:4)* \| Phospholipid Metabolism \| 0.07 \| 0.39 \| \| linoleoyl-arachidonoyl-glycerol (18:2/20:4) [2]* \| Diacylglycerol \| 0.07 \| -0.05 \| \| alpha-hydroxyisovalerate \| Leucine, Isoleucine and Valine Metabolism \| 0.07 \| -0.05 \| \| palmitoyl-myristoyl-glycerol (16:0/14:0) [2] \| Diacylglycerol \| 0.06 \| 0.37 \| \| linoleoyl-linolenoyl-glycerol (18:2/18:3) [2]* \| Diacylglycerol \| 0.06 \| 0.06 \| \| myristoylcarnitine (C14) \| Fatty Acid Metabolism(Acyl Carnitine) \| 0.06 \| 0.17 \| \| isoursodeoxycholate \| Secondary Bile Acid Metabolism \| 0.05 \| -0.02 \| \| 3-carboxy-4-methyl-5-propyl-2-furanpropanoate (CMPF) \| Fatty Acid, Dicarboxylate \| 0.05 \| -0.24 \| \| 2-stearoyl-GPE (18:0)* \| Lysolipid \| 0.05 \| 0.37 \| \| palmitoyl-arachidonoyl-glycerol (16:0/20:4) [2]* \| Diacylglycerol \| 0.05 \| 0.47 \| \| myristoleoylcarnitine (C14:1)* \| Fatty Acid Metabolism(Acyl Carnitine) \| 0.05 \| 0.17 \| \| linoleoyl-linolenoyl-glycerol (18:2/18:3) [1]* \| Diacylglycerol \| 0.05 \| -0.03 \| \| sphingomyelin (d18:1/20:1, d18:2/20:0)* \| Sphingolipid Metabolism \| 0.04 \| -0.04 \| \| 1-oleoylglycerol (18:1) \| Monoacylglycerol \| 0.04 \| -0.19 \| \| sphingomyelin (d17:1/16:0, d18:1/15:0, d16:1/17:0)* \| Sphingolipid Metabolism \| 0.04 \| 0.33 \| \| palmitoleoylcarnitine (C16:1)* \| Fatty Acid Metabolism(Acyl Carnitine) \| 0.04 \| 0.04 \| \| sphingomyelin (d18:1/14:0, d16:1/16:0)* \| Sphingolipid Metabolism \| 0.04 \| 0.20 \| \| succinylcarnitine (C4-DC) \| TCA Cycle \| 0.04 \| 0.31 \| \| glycosyl-N-stearoyl-sphingosine (d18:1/18:0) \| Sphingolipid Metabolism \| 0.03 \| 0.07 \| \| methionine sulfoxide \| Methionine, Cysteine, SAM and Taurine Metabolism \| 0.03 \| -0.19 \| \| erythritol \| Food Component/Plant \| 0.03 \| 0.07 \| \| 1-palmitoyl-GPE (16:0) \| Lysolipid \| 0.03 \| 0.62 \| \| diacylglycerol (14:0/18:1, 16:0/16:1) [2]* \| Diacylglycerol \| 0.03 \| 0.44 \| \| 1-stearoyl-2-oleoyl-GPI (18:0/18:1)* \| Phospholipid Metabolism \| 0.03 \| -0.09 \| \| docosahexaenoate (DHA; 22:6n3) \| Polyunsaturated Fatty Acid (n3 and n6) \| 0.02 \| 0.12 \| \| behenoyl dihydrosphingomyelin (d18:0/22:0)* \| Sphingolipid Metabolism \| 0.02 \| 0.37 \| \| sphingomyelin (d18:2/16:0, d18:1/16:1)* \| Sphingolipid Metabolism \| 0.02 \| 0.17 \| \| citrulline \| Urea cycle; Arginine and Proline Metabolism \| 0.02 \| -0.03 \| \| diacylglycerol (14:0/18:1, 16:0/16:1) [1]* \| Diacylglycerol \| 0.02 \| 0.43 \| \| sphingomyelin (d18:2/21:0, d16:2/23:0)* \| Sphingolipid Metabolism \| 0.02 \| 0.29 \| \| sphingomyelin (d18:1/19:0, d19:1/18:0)* \| Sphingolipid Metabolism \| 0.02 \| 0.42 \| \| 1-linoleoylglycerol (18:2) \| Monoacylglycerol \| 0.02 \| -0.47 \| \| 1-palmitoyl-2-oleoyl-GPC (16:0/18:1) \| Phospholipid Metabolism \| 0.02 \| 0.24 \| \| dihomo-linoleate (20:2n6) \| Polyunsaturated Fatty Acid (n3 and n6) \| 0.02 \| -0.03 \| \| pelargonate (9:0) \| Medium Chain Fatty Acid \| 0.01 \| -0.06 \| \| piperine \| Food Component/Plant \| 0.01 \| 0.10 \| \| 2-methylbutyrylcarnitine (C5) \| Leucine, Isoleucine and Valine Metabolism \| 0.01 \| 0.09 \| \| saccharin \| Food Component/Plant \| 0.01 \| -0.21 \| \| p-cresol-glucuronide* \| Phenylalanine and Tyrosine Metabolism \| 0.01 \| 0.15 \| \| betaine \| Glycine, Serine and Threonine Metabolism \| 0.01 \| -0.19 \| \| 1-palmitoleoyl-2-linoleoyl-GPC (16:1/18:2)* \| Phospholipid Metabolism \| 0.01 \| -0.02 \| \| 3-indoxyl sulfate \| Tryptophan Metabolism \| 0.00 \| -0.18 \| \| oleoyl-oleoyl-glycerol (18:1/18:1) [2]* \| Diacylglycerol \| 0.00 \| 0.50 \| \| glycolithocholate sulfate* \| Secondary Bile Acid Metabolism \| 0.00 \| -0.01 \| \| glycoursodeoxycholate \| Secondary Bile Acid Metabolism \| 0.00 \| 0.05 \| \| 3-methoxytyrosine \| Phenylalanine and Tyrosine Metabolism \| 0.00 \| -0.08 \| \| 1-linoleoyl-GPA (18:2)* \| Lysolipid \| 0.00 \| -0.31 \| \| 2-palmitoylglycerol (16:0) \| Monoacylglycerol \| 0.00 \| -0.21 \| \| 1-stearoyl-GPE (18:0) \| Lysolipid \| 0.00 \| 0.61 \| \| linoleate (18:2n6) \| Polyunsaturated Fatty Acid (n3 and n6) \| 0.00 \| 0.03 \| \| proline \| Urea cycle; Arginine and Proline Metabolism \| 0.00 \| -0.01 \| \| 1-palmitoyl-2-linoleoyl-GPE (16:0/18:2) \| Phospholipid Metabolism \| 0.00 \| 0.55 \| \| picolinate \| Tryptophan Metabolism \| 0.00 \| 0.28 \| \| N-acetylvaline \| Leucine, Isoleucine and Valine Metabolism \| -0.61 \| 0.16 \| \| N-acetylleucine \| Leucine, Isoleucine and Valine Metabolism \| -0.54 \| -0.04 \| \| N-acetylglutamine \| Glutamate Metabolism \| -0.52 \| -0.30 \| \| gamma-glutamylthreonine \| Gamma-glutamyl Amino Acid \| -0.45 \| -0.15 \| \| 3-(4-hydroxyphenyl)lactate \| Phenylalanine and Tyrosine Metabolism \| -0.45 \| -0.22 \| \| 2-hydroxyoctanoate \| Fatty Acid, Monohydroxy \| -0.44 \| -0.16 \| \| indolelactate \| Tryptophan Metabolism \| -0.42 \| -0.20 \| \| gamma-glutamylleucine \| Gamma-glutamyl Amino Acid \| -0.42 \| -0.13 \| \| gamma-glutamyltyrosine \| Gamma-glutamyl Amino Acid \| -0.42 \| -0.20 \| \| 2-keto-3-deoxy-gluconate \| Food Component/Plant \| -0.41 \| 0.07 \| \| N-acetylisoleucine \| Leucine, Isoleucine and Valine Metabolism \| -0.41 \| -0.17 \| \| gamma-glutamyl-epsilon-lysine \| Gamma-glutamyl Amino Acid \| -0.40 \| -0.02 \| \| gamma-glutamylvaline \| Gamma-glutamyl Amino Acid \| -0.39 \| 0.13 \| \| 13-HODE + 9-HODE \| Fatty Acid, Monohydroxy \| -0.39 \| 0.08 \| \| 1-oleoyl-GPS (18:1) \| Lysolipid \| -0.39 \| -0.04 \| \| cysteine \| Methionine, Cysteine, SAM and Taurine Metabolism \| -0.38 \| -0.03 \| \| imidazole lactate \| Histidine Metabolism \| -0.38 \| -0.09 \| \| alliin \| Food Component/Plant \| -0.38 \| -0.09 \| \| valine \| Leucine, Isoleucine and Valine Metabolism \| -0.37 \| 0.13 \| \| tryptophan \| Tryptophan Metabolism \| -0.36 \| -0.15 \| \| N-acetylarginine \| Urea cycle; Arginine and Proline Metabolism \| -0.36 \| 0.07 \| \| thymol sulfate \| Food Component/Plant \| -0.36 \| 0.26 \| \| 5-acetylamino-6-formylamino-3-methyluracil \| Xanthine Metabolism \| -0.36 \| -0.19 \| \| trans-urocanate \| Histidine Metabolism \| -0.35 \| -0.07 \| \| pyroglutamine* \| Glutamate Metabolism \| -0.35 \| -0.23 \| \| 3-methylxanthine \| Xanthine Metabolism \| -0.35 \| 0.22 \| \| trans-4-hydroxyproline \| Urea cycle; Arginine and Proline Metabolism \| -0.35 \| 0.24 \| \| N-acetylglutamate \| Glutamate Metabolism \| -0.35 \| 0.45 \| \| N-acetylphenylalanine \| Phenylalanine and Tyrosine Metabolism \| -0.34 \| -0.44 \| \| glycerophosphorylcholine (GPC) \| Phospholipid Metabolism \| -0.34 \| 0.18 \| \| glycerophosphoethanolamine \| Phospholipid Metabolism \| -0.34 \| 0.27 \| \| N-formylmethionine \| Methionine, Cysteine, SAM and Taurine Metabolism \| -0.34 \| 0.17 \| \| gamma-glutamylisoleucine* \| Gamma-glutamyl Amino Acid \| -0.34 \| 0.00 \| \| N-acetylhistidine \| Histidine Metabolism \| -0.33 \| -0.05 \| \| theobromine \| Xanthine Metabolism \| -0.33 \| -0.08 \| \| kynurenate \| Tryptophan Metabolism \| -0.32 \| -0.19 \| \| glutamate \| Glutamate Metabolism \| -0.32 \| -0.03 \| \| threonine \| Glycine, Serine and Threonine Metabolism \| -0.32 \| -0.13 \| \| 5alpha-androstan-3beta,17beta-diol monosulfate (2) \| Steroid \| -0.32 \| -0.33 \| \| 1-linoleoyl-GPG (18:2)* \| Lysolipid \| -0.32 \| -0.24 \| \| glutamine \| Glutamate Metabolism \| -0.31 \| -0.02 \| \| 1,7-dimethylurate \| Xanthine Metabolism \| -0.31 \| 0.04 \| \| choline phosphate \| Phospholipid Metabolism \| -0.31 \| 0.04 \| \| malate \| TCA Cycle \| -0.31 \| 0.10 \| \| 2-hydroxyglutarate \| Fatty Acid, Dicarboxylate \| -0.31 \| 0.13 \| \| cortisol \| Steroid \| -0.31 \| 0.38 \| \| paraxanthine \| Xanthine Metabolism \| -0.30 \| 0.06 \| \| homoarginine \| Urea cycle; Arginine and Proline Metabolism \| -0.30 \| -0.20 \| \| 5alpha-androstan-3alpha,17beta-diol monosulfate (1) \| Steroid \| -0.30 \| -0.27 \| \| N-acetylserine \| Glycine, Serine and Threonine Metabolism \| -0.30 \| 0.32 \| \| N-acetyltyrosine \| Phenylalanine and Tyrosine Metabolism \| -0.30 \| -0.17 \| \| gamma-glutamyl-alpha-lysine \| Gamma-glutamyl Amino Acid \| -0.30 \| -0.02 \| \| dimethylarginine (SDMA + ADMA) \| Urea cycle; Arginine and Proline Metabolism \| -0.29 \| 0.15 \| \| 1,3,7-trimethylurate \| Xanthine Metabolism \| -0.29 \| -0.15 \| \| 1-linoleoyl-GPE (18:2)* \| Lysolipid \| -0.29 \| -0.06 \| \| 1,2-dilinoleoyl-GPC (18:2/18:2) \| Phospholipid Metabolism \| -0.29 \| -0.16 \| \| 1-methylhistidine \| Histidine Metabolism \| -0.29 \| 0.04 \| \| 1-stearoyl-2-linoleoyl-GPC (18:0/18:2)* \| Phospholipid Metabolism \| -0.29 \| 0.03 \| \| N-behenoyl-sphingadienine (d18:2/22:0)* \| Sphingolipid Metabolism \| -0.29 \| 0.18 \| \| 1-methylxanthine \| Xanthine Metabolism \| -0.29 \| 0.19 \| \| S-adenosylhomocysteine (SAH) \| Methionine, Cysteine, SAM and Taurine Metabolism \| -0.29 \| -0.02 \| \| 2-methylcitrate/homocitrate \| TCA Cycle \| -0.28 \| -0.10 \| \| alpha-hydroxyisocaproate \| Leucine, Isoleucine and Valine Metabolism \| -0.28 \| 0.03 \| \| lactosyl-N-nervonoyl-sphingosine (d18:1/24:1)* \| Sphingolipid Metabolism \| -0.28 \| -0.17 \| \| urea \| Urea cycle; Arginine and Proline Metabolism \| -0.28 \| 0.40 \| \| gamma-glutamylhistidine \| Gamma-glutamyl Amino Acid \| -0.28 \| -0.21 \| \| 3-hydroxyhippurate \| Benzoate Metabolism \| -0.27 \| -0.09 \| \| 3-methyl-2-oxovalerate \| Leucine, Isoleucine and Valine Metabolism \| -0.27 \| 0.14 \| \| adipate \| Fatty Acid, Dicarboxylate \| -0.27 \| 0.10 \| \| propyl 4-hydroxybenzoate sulfate \| Benzoate Metabolism \| -0.27 \| 0.03 \| \| glycosyl-N-palmitoyl-sphingosine (d18:1/16:0) \| Sphingolipid Metabolism \| -0.27 \| 0.33 \| \| citrate \| TCA Cycle \| -0.27 \| -0.23 \| \| lignoceroyl sphingomyelin (d18:1/24:0) \| Sphingolipid Metabolism \| -0.26 \| 0.06 \| \| androstenediol (3alpha, 17alpha) monsulfate (2) \| Steroid \| -0.26 \| -0.20 \| \| behenoyl sphingomyelin (d18:1/22:0)* \| Sphingolipid Metabolism \| -0.26 \| 0.28 \| \| 4-hydroxyphenylpyruvate \| Phenylalanine and Tyrosine Metabolism \| -0.26 \| 0.39 \| \| succinate \| TCA Cycle \| -0.26 \| 0.28 \| \| 1-arachidonoyl-GPE (20:4n6)* \| Lysolipid \| -0.26 \| 0.08 \| \| S-methylcysteine \| Methionine, Cysteine, SAM and Taurine Metabolism \| -0.26 \| -0.35 \| \| N-acetyl-1-methylhistidine* \| Histidine Metabolism \| -0.26 \| 0.12 \| \| octadecanedioate \| Fatty Acid, Dicarboxylate \| -0.25 \| 0.42 \| \| fumarate \| TCA Cycle \| -0.25 \| 0.08 \| \| 2-hydroxydecanoate \| Fatty Acid, Monohydroxy \| -0.25 \| -0.11 \| \| sarcosine \| Glycine, Serine and Threonine Metabolism \| -0.25 \| 0.01 \| \| 5-bromotryptophan \| Phenylalanine and Tyrosine Metabolism \| -0.25 \| -0.34 \| \| hypotaurine \| Methionine, Cysteine, SAM and Taurine Metabolism \| -0.25 \| 0.04 \| \| 1-arachidonoyl-GPI (20:4)* \| Lysolipid \| -0.25 \| -0.11 \| \| androstenediol (3beta,17beta) disulfate (2) \| Steroid \| -0.25 \| -0.20 \| \| taurine \| Methionine, Cysteine, SAM and Taurine Metabolism \| -0.24 \| -0.07 \| \| phenylalanine \| Phenylalanine and Tyrosine Metabolism \| -0.24 \| 0.01 \| \| 3-methyl-2-oxobutyrate \| Leucine, Isoleucine and Valine Metabolism \| -0.24 \| 0.15 \| \| cerotoylcarnitine (C26)* \| Fatty Acid Metabolism(Acyl Carnitine) \| -0.24 \| 0.17 \| \| 5alpha-androstan-3beta,17alpha-diol disulfate \| Steroid \| -0.24 \| -0.49 \| \| 16-hydroxypalmitate \| Fatty Acid, Monohydroxy \| -0.24 \| 0.34 \| \| leucine \| Leucine, Isoleucine and Valine Metabolism \| -0.24 \| 0.11 \| \| tyrosine \| Phenylalanine and Tyrosine Metabolism \| -0.23 \| 0.09 \| \| 8-hydroxyoctanoate \| Fatty Acid, Monohydroxy \| -0.23 \| 0.20 \| \| 1-linoleoyl-GPI (18:2)* \| Lysolipid \| -0.23 \| 0.03 \| \| maleate \| Fatty Acid, Dicarboxylate \| -0.23 \| 0.58 \| \| 3-hydroxylaurate \| Fatty Acid, Monohydroxy \| -0.23 \| 0.59 \| \| glycohyocholate \| Secondary Bile Acid Metabolism \| -0.23 \| 0.11 \| \| trimethylamine N-oxide \| Phospholipid Metabolism \| -0.23 \| 0.29 \| \| stearoylcarnitine (C18) \| Fatty Acid Metabolism(Acyl Carnitine) \| -0.23 \| 0.19 \| \| gamma-glutamylphenylalanine \| Gamma-glutamyl Amino Acid \| -0.22 \| -0.18 \| \| azelate (nonanedioate) \| Fatty Acid, Dicarboxylate \| -0.22 \| -0.20 \| \| isovalerylglycine \| Leucine, Isoleucine and Valine Metabolism \| -0.22 \| -0.07 \| \| catechol sulfate \| Benzoate Metabolism \| -0.22 \| -0.07 \| \| theophylline \| Xanthine Metabolism \| -0.22 \| -0.03 \| \| 5alpha-androstan-3alpha,17beta-diol disulfate \| Steroid \| -0.22 \| -0.01 \| \| nervonate (24:1n9)* \| Long Chain Fatty Acid \| -0.22 \| 0.18 \| \| 1-(1-enyl-palmitoyl)-2-arachidonoyl-GPC (P-16:0/20:4)* \| Plasmalogen \| -0.22 \| -0.10 \| \| eicosanodioate \| Fatty Acid, Dicarboxylate \| -0.22 \| -0.25 \| \| o-cresol sulfate \| Phenylalanine and Tyrosine Metabolism \| -0.21 \| 0.06 \| \| C-glycosyltryptophan \| Tryptophan Metabolism \| -0.21 \| -0.19 \| \| behenate (22:0)* \| Long Chain Fatty Acid \| -0.21 \| -0.25 \| \| sphinganine-1-phosphate \| Sphingolipid Metabolism \| -0.21 \| -0.09 \| \| dihomo-linoleoylcarnitine (C20:2)* \| Fatty Acid Metabolism(Acyl Carnitine) \| -0.21 \| 0.00 \| \| S-methylcysteine sulfoxide \| Methionine, Cysteine, SAM and Taurine Metabolism \| -0.21 \| -0.09 \| \| etiocholanolone glucuronide \| Steroid \| -0.21 \| 0.07 \| \| quinate \| Food Component/Plant \| -0.20 \| -0.11 \| \| N-acetylmethionine \| Methionine, Cysteine, SAM and Taurine Metabolism \| -0.20 \| 0.01 \| \| indoleacetate \| Tryptophan Metabolism \| -0.20 \| -0.01 \| \| formiminoglutamate \| Histidine Metabolism \| -0.20 \| 0.33 \| \| caffeine \| Xanthine Metabolism \| -0.20 \| -0.01 \| \| sphingomyelin (d18:2/14:0, d18:1/14:1)* \| Sphingolipid Metabolism \| -0.20 \| -0.07 \| \| docosadioate \| Fatty Acid, Dicarboxylate \| -0.20 \| -0.13 \| \| dimethylglycine \| Glycine, Serine and Threonine Metabolism \| -0.20 \| 0.18 \| \| N-acetyltryptophan \| Tryptophan Metabolism \| -0.20 \| -0.19 \| \| 7-methylurate \| Xanthine Metabolism \| -0.20 \| 0.18 \| \| docosapentaenoate (n6 DPA; 22:5n6) \| Polyunsaturated Fatty Acid (n3 and n6) \| -0.20 \| 0.04 \| \| hyocholate \| Secondary Bile Acid Metabolism \| -0.20 \| 0.12 \| \| 3-methyl catechol sulfate (1) \| Benzoate Metabolism \| -0.19 \| 0.03 \| \| androstenediol (3beta,17beta) disulfate (1) \| Steroid \| -0.19 \| -0.28 \| \| 1-stearoyl-2-linoleoyl-GPI (18:0/18:2) \| Phospholipid Metabolism \| -0.19 \| 0.11 \| \| 1-(1-enyl-palmitoyl)-2-linoleoyl-GPE (P-16:0/18:2)* \| Plasmalogen \| -0.19 \| 0.33 \| \| O-methylcatechol sulfate \| Benzoate Metabolism \| -0.19 \| 0.08 \| \| N-acetyltaurine \| Methionine, Cysteine, SAM and Taurine Metabolism \| -0.19 \| -0.36 \| \| androsterone sulfate \| Steroid \| -0.19 \| -0.36 \| \| argininosuccinate \| Urea cycle; Arginine and Proline Metabolism \| -0.19 \| 0.43 \| \| pregnen-diol disulfate* \| Steroid \| -0.19 \| -0.22 \| \| 1-oleoyl-GPE (18:1) \| Lysolipid \| -0.18 \| 0.15 \| \| ximenoylcarnitine (C26:1)* \| Fatty Acid Metabolism(Acyl Carnitine) \| -0.18 \| 0.04 \| \| docosapentaenoylcarnitine (C22:5n3)* \| Fatty Acid Metabolism(Acyl Carnitine) \| -0.18 \| 0.00 \| \| myristoleate (14:1n5) \| Long Chain Fatty Acid \| -0.18 \| 0.30 \| \| 1-linoleoyl-2-linolenoyl-GPC (18:2/18:3)* \| Phospholipid Metabolism \| -0.18 \| 0.07 \| \| androstenediol (3beta,17beta) monosulfate (2) \| Steroid \| -0.18 \| -0.38 \| \| 1-(1-enyl-palmitoyl)-2-palmitoyl-GPC (P-16:0/16:0)* \| Plasmalogen \| -0.18 \| 0.04 \| \| eugenol sulfate \| Food Component/Plant \| -0.18 \| 0.39 \| \| 1-linoleoyl-GPC (18:2) \| Lysolipid \| -0.18 \| -0.28 \| \| cysteine s-sulfate \| Methionine, Cysteine, SAM and Taurine Metabolism \| -0.18 \| -0.07 \| \| methyl-4-hydroxybenzoate sulfate \| Benzoate Metabolism \| -0.18 \| -0.09 \| \| N-alpha-acetylornithine \| Urea cycle; Arginine and Proline Metabolism \| -0.18 \| -0.02 \| \| tricosanoyl sphingomyelin (d18:1/23:0)* \| Sphingolipid Metabolism \| -0.17 \| 0.34 \| \| arachidoylcarnitine (C20)* \| Fatty Acid Metabolism(Acyl Carnitine) \| -0.17 \| 0.19 \| \| linoleoylcarnitine (C18:2)* \| Fatty Acid Metabolism(Acyl Carnitine) \| -0.17 \| -0.11 \| \| 1-linoleoyl-2-arachidonoyl-GPC (18:2/20:4n6)* \| Phospholipid Metabolism \| -0.17 \| 0.01 \| \| lignoceroylcarnitine (C24)* \| Fatty Acid Metabolism(Acyl Carnitine) \| -0.17 \| -0.04 \| \| 1,3-dimethylurate \| Xanthine Metabolism \| -0.17 \| -0.09 \| \| palmitoyl-palmitoyl-glycerol (16:0/16:0) [1]* \| Diacylglycerol \| -0.17 \| 0.45 \| \| androstenediol (3beta,17beta) monosulfate (1) \| Steroid \| -0.17 \| -0.14 \| \| pregn steroid monosulfate* \| Steroid \| -0.17 \| -0.28 \| \| 5alpha-androstan-3beta,17beta-diol disulfate \| Steroid \| -0.17 \| -0.34 \| \| N-acetylthreonine \| Glycine, Serine and Threonine Metabolism \| -0.17 \| 0.11 \| \| 1-methylimidazoleacetate \| Histidine Metabolism \| -0.17 \| 0.06 \| \| stearate (18:0) \| Long Chain Fatty Acid \| -0.16 \| 0.14 \| \| 2-oleoylglycerol (18:1) \| Monoacylglycerol \| -0.16 \| -0.08 \| \| 3-hydroxydecanoate \| Fatty Acid, Monohydroxy \| -0.16 \| 0.69 \| \| cortisone \| Steroid \| -0.16 \| -0.22 \| \| epiandrosterone sulfate \| Steroid \| -0.16 \| -0.36 \| \| 1-palmitoyl-2-stearoyl-GPC (16:0/18:0) \| Phospholipid Metabolism \| -0.15 \| 0.41 \| \| sphingomyelin (d18:1/24:1, d18:2/24:0)* \| Sphingolipid Metabolism \| -0.15 \| -0.09 \| \| caprylate (8:0) \| Medium Chain Fatty Acid \| -0.15 \| -0.36 \| \| thyroxine \| Phenylalanine and Tyrosine Metabolism \| -0.15 \| -0.17 \| \| 5-acetylamino-6-amino-3-methyluracil \| Xanthine Metabolism \| -0.15 \| 0.02 \| \| stearidonate (18:4n3) \| Polyunsaturated Fatty Acid (n3 and n6) \| -0.15 \| 0.25 \| \| dopamine 3-O-sulfate \| Phenylalanine and Tyrosine Metabolism \| -0.15 \| -0.27 \| \| methionine \| Methionine, Cysteine, SAM and Taurine Metabolism \| -0.15 \| -0.24 \| \| sphingomyelin (d18:1/22:1, d18:2/22:0, d16:1/24:1)* \| Sphingolipid Metabolism \| -0.15 \| 0.06 \| \| erucate (22:1n9) \| Long Chain Fatty Acid \| -0.15 \| 0.01 \| \| N-(2-furoyl)glycine \| Food Component/Plant \| -0.15 \| -0.23 \| \| beta-citrylglutamate \| Glutamate Metabolism \| -0.15 \| 0.05 \| \| 1-stearoyl-2-arachidonoyl-GPE (18:0/20:4) \| Phospholipid Metabolism \| -0.15 \| 0.35 \| \| 4-methyl-2-oxopentanoate \| Leucine, Isoleucine and Valine Metabolism \| -0.15 \| 0.19 \| \| N-formylphenylalanine \| Phenylalanine and Tyrosine Metabolism \| -0.15 \| -0.02 \| \| 1-palmitoyl-2-linoleoyl-GPC (16:0/18:2) \| Phospholipid Metabolism \| -0.14 \| 0.26 \| \| andro steroid monosulfate (1)* \| Steroid \| -0.14 \| -0.30 \| \| S-1-pyrroline-5-carboxylate \| Glutamate Metabolism \| -0.14 \| -0.07 \| \| 5-dodecenoate (12:1n7) \| Medium Chain Fatty Acid \| -0.14 \| 0.34 \| \| serine \| Glycine, Serine and Threonine Metabolism \| -0.14 \| -0.45 \| \| adrenoylcarnitine (C22:4)* \| Fatty Acid Metabolism(Acyl Carnitine) \| -0.14 \| -0.07 \| \| sphingomyelin (d18:1/25:0, d19:0/24:1, d20:1/23:0, d19:1/24:0)* \| Sphingolipid Metabolism \| -0.14 \| 0.19 \| \| 1-oleoyl-GPI (18:1)* \| Lysolipid \| -0.14 \| -0.12 \| \| dihomo-linolenate (20:3n3 or n6) \| Polyunsaturated Fatty Acid (n3 and n6) \| -0.14 \| -0.19 \| \| 2-hydroxy-3-methylvalerate \| Leucine, Isoleucine and Valine Metabolism \| -0.14 \| -0.02 \| \| ornithine \| Urea cycle; Arginine and Proline Metabolism \| -0.14 \| -0.14 \| \| imidazole propionate \| Histidine Metabolism \| -0.13 \| 0.23 \| \| eicosenoylcarnitine (C20:1)* \| Fatty Acid Metabolism(Acyl Carnitine) \| -0.13 \| 0.11 \| \| 1-stearoyl-GPI (18:0) \| Lysolipid \| -0.13 \| -0.20 \| \| docosadienoate (22:2n6) \| Polyunsaturated Fatty Acid (n3 and n6) \| -0.13 \| 0.10 \| \| 1-(1-enyl-stearoyl)-2-arachidonoyl-GPE (P-18:0/20:4)* \| Plasmalogen \| -0.13 \| 0.44 \| \| acetylcarnitine (C2) \| Fatty Acid Metabolism(Acyl Carnitine) \| -0.13 \| 0.45 \| \| arachidate (20:0) \| Long Chain Fatty Acid \| -0.13 \| -0.08 \| \| 2-hydroxyhippurate (salicylurate) \| Benzoate Metabolism \| -0.13 \| 0.29 \| \| glycine \| Glycine, Serine and Threonine Metabolism \| -0.13 \| -0.10 \| \| 5-hydroxymethyl-2-furoic acid \| Phenylalanine and Tyrosine Metabolism \| -0.13 \| -0.18 \| \| 2-hydroxystearate \| Fatty Acid, Monohydroxy \| -0.13 \| -0.03 \| \| gamma-glutamyltryptophan \| Gamma-glutamyl Amino Acid \| -0.13 \| -0.21 \| \| 1-(1-enyl-palmitoyl)-2-linoleoyl-GPC (P-16:0/18:2)* \| Plasmalogen \| -0.12 \| -0.09 \| \| sphingosine 1-phosphate \| Sphingolipid Metabolism \| -0.12 \| -0.29 \| \| palmitoyl-oleoyl-glycerol (16:0/18:1) [1]* \| Diacylglycerol \| -0.12 \| 0.63 \| \| 1-(1-enyl-palmitoyl)-2-oleoyl-GPC (P-16:0/18:1)* \| Plasmalogen \| -0.12 \| -0.05 \| \| histidine \| Histidine Metabolism \| -0.12 \| 0.00 \| \| sphingomyelin (d18:2/23:0, d18:1/23:1, d17:1/24:1)* \| Sphingolipid Metabolism \| -0.12 \| 0.10 \| \| glycodeoxycholate \| Secondary Bile Acid Metabolism \| -0.12 \| 0.25 \| \| phenol sulfate \| Phenylalanine and Tyrosine Metabolism \| -0.12 \| -0.37 \| \| hippurate \| Benzoate Metabolism \| -0.12 \| 0.20 \| \| octanoylcarnitine (C8) \| Fatty Acid Metabolism(Acyl Carnitine) \| -0.12 \| 0.44 \| \| cinnamoylglycine \| Food Component/Plant \| -0.12 \| -0.10 \| \| margaroylcarnitine* \| Fatty Acid Metabolism(Acyl Carnitine) \| -0.12 \| 0.25 \| \| 1,2-dipalmitoyl-GPC (16:0/16:0) \| Phospholipid Metabolism \| -0.12 \| 0.40 \| \| linolenate [alpha or gamma; (18:3n3 or 6)] \| Polyunsaturated Fatty Acid (n3 and n6) \| -0.11 \| 0.09 \| \| 3-hydroxystearate \| Fatty Acid, Monohydroxy \| -0.11 \| 0.19 \| \| androstenediol (3alpha, 17alpha) monsulfate (3) \| Steroid \| -0.11 \| -0.32 \| \| alpha-ketoglutarate \| TCA Cycle \| -0.11 \| 0.22 \| \| argininate* \| Urea cycle; Arginine and Proline Metabolism \| -0.11 \| 0.36 \| \| eicosenoate (20:1) \| Long Chain Fatty Acid \| -0.11 \| 0.11 \| \| 1-stearoyl-2-arachidonoyl-GPC (18:0/20:4) \| Phospholipid Metabolism \| -0.11 \| -0.19 \| \| oleate/vaccenate (18:1) \| Long Chain Fatty Acid \| -0.11 \| 0.22 \| \| phenyllactate (PLA) \| Phenylalanine and Tyrosine Metabolism \| -0.11 \| -0.07 \| \| myristate (14:0) \| Long Chain Fatty Acid \| -0.11 \| 0.20 \| \| arachidonoylcarnitine (C20:4) \| Fatty Acid Metabolism(Acyl Carnitine) \| -0.11 \| -0.07 \| \| oleoylcarnitine (C18:1) \| Fatty Acid Metabolism(Acyl Carnitine) \| -0.11 \| -0.14 \| \| 1-(1-enyl-palmitoyl)-2-arachidonoyl-GPE (P-16:0/20:4)* \| Plasmalogen \| -0.11 \| 0.21 \| \| N-palmitoyl-sphinganine (d18:0/16:0) \| Sphingolipid Metabolism \| -0.11 \| 0.45 \| \| sphingosine \| Sphingolipid Metabolism \| -0.11 \| -0.22 \| \| 1-linolenoyl-GPC (18:3)* \| Lysolipid \| -0.10 \| -0.03 \| \| N-acetylglycine \| Glycine, Serine and Threonine Metabolism \| -0.10 \| 0.01 \| \| hexadecanedioate \| Fatty Acid, Dicarboxylate \| -0.10 \| 0.51 \| \| methylsuccinate \| Leucine, Isoleucine and Valine Metabolism \| -0.10 \| 0.25 \| \| N-stearoyl-sphingosine (d18:1/18:0)* \| Sphingolipid Metabolism \| -0.10 \| 0.62 \| \| pro-hydroxy-pro \| Urea cycle; Arginine and Proline Metabolism \| -0.10 \| 0.19 \| \| taurodeoxycholate \| Secondary Bile Acid Metabolism \| -0.10 \| 0.14 \| \| 1-stearoyl-2-arachidonoyl-GPI (18:0/20:4) \| Phospholipid Metabolism \| -0.10 \| -0.20 \| \| 1-palmitoyl-2-arachidonoyl-GPI (16:0/20:4)* \| Phospholipid Metabolism \| -0.10 \| 0.00 \| \| palmitoyl-oleoyl-glycerol (16:0/18:1) [2]* \| Diacylglycerol \| -0.10 \| 0.64 \| \| methionine sulfone \| Methionine, Cysteine, SAM and Taurine Metabolism \| -0.10 \| -0.06 \| \| phosphoethanolamine \| Phospholipid Metabolism \| -0.10 \| 0.23 \| \| 1-stearoyl-GPS (18:0)* \| Lysolipid \| -0.10 \| -0.14 \| \| 1-dihomo-linolenylglycerol (20:3) \| Monoacylglycerol \| -0.09 \| -0.49 \| \| palmitoyl dihydrosphingomyelin (d18:0/16:0)* \| Sphingolipid Metabolism \| -0.09 \| 0.31 \| \| 2-ethylphenylsulfate \| Benzoate Metabolism \| -0.09 \| 0.09 \| \| cis-aconitate \| TCA Cycle \| -0.09 \| -0.15 \| \| palmitoleate (16:1n7) \| Long Chain Fatty Acid \| -0.09 \| 0.19 \| \| dihomo-linolenoylcarnitine (20:3n3 or 6)* \| Fatty Acid Metabolism(Acyl Carnitine) \| -0.09 \| -0.11 \| \| decanoylcarnitine (C10) \| Fatty Acid Metabolism(Acyl Carnitine) \| -0.09 \| 0.34 \| \| 3-methoxycatechol sulfate (1) \| Benzoate Metabolism \| -0.09 \| 0.21 \| \| ergothioneine \| Food Component/Plant \| -0.09 \| -0.12 \| \| heptanoate (7:0) \| Medium Chain Fatty Acid \| -0.09 \| 0.27 \| \| N-palmitoyl-sphingosine (d18:1/16:0) \| Sphingolipid Metabolism \| -0.09 \| 0.41 \| \| 10-nonadecenoate (19:1n9) \| Long Chain Fatty Acid \| -0.09 \| 0.24 \| \| taurocholenate sulfate \| Secondary Bile Acid Metabolism \| -0.09 \| 0.14 \| \| stearoyl-arachidonoyl-glycerol (18:0/20:4) [2]* \| Diacylglycerol \| -0.09 \| 0.48 \| \| 4-vinylphenol sulfate \| Benzoate Metabolism \| -0.08 \| -0.06 \| \| 1-stearoyl-GPC (18:0) \| Lysolipid \| -0.08 \| 0.06 \| \| 1-stearoyl-2-oleoyl-GPC (18:0/18:1) \| Phospholipid Metabolism \| -0.08 \| 0.26 \| \| glycodeoxycholate sulfate \| Secondary Bile Acid Metabolism \| -0.08 \| 0.27 \| \| 1-stearoyl-2-oleoyl-GPE (18:0/18:1) \| Phospholipid Metabolism \| -0.08 \| 0.37 \| \| 1-(1-enyl-palmitoyl)-2-oleoyl-GPE (P-16:0/18:1)* \| Plasmalogen \| -0.08 \| 0.20 \| \| nonadecanoate (19:0) \| Long Chain Fatty Acid \| -0.08 \| 0.00 \| \| gamma-glutamylmethionine \| Gamma-glutamyl Amino Acid \| -0.08 \| -0.26 \| \| oleoyl-oleoyl-glycerol (18:1/18:1) [1]* \| Diacylglycerol \| -0.08 \| 0.44 \| \| arachidonate (20:4n6) \| Polyunsaturated Fatty Acid (n3 and n6) \| -0.08 \| -0.11 \| \| N-acetylproline \| Urea cycle; Arginine and Proline Metabolism \| -0.07 \| 0.00 \| \| palmitoyl-palmitoyl-glycerol (16:0/16:0) [2]* \| Diacylglycerol \| -0.07 \| 0.54 \| \| 2-hydroxybutyrate/2-hydroxyisobutyrate \| Methionine, Cysteine, SAM and Taurine Metabolism \| -0.07 \| 0.40 \| \| 14-HDoHE/17-HDoHE \| Fatty Acid, Monohydroxy \| -0.07 \| 0.25 \| \| palmitoylcarnitine (C16) \| Fatty Acid Metabolism(Acyl Carnitine) \| -0.07 \| 0.05 \| \| 16a-hydroxy DHEA 3-sulfate \| Steroid \| -0.07 \| -0.22 \| \| 2-hydroxypalmitate \| Fatty Acid, Monohydroxy \| -0.07 \| 0.11 \| \| glycerophosphoinositol* \| Phospholipid Metabolism \| -0.07 \| 0.00 \| \| 3-hydroxyisobutyrate \| Leucine, Isoleucine and Valine Metabolism \| -0.07 \| -0.20 \| \| laurylcarnitine (C12) \| Fatty Acid Metabolism(Acyl Carnitine) \| -0.06 \| 0.31 \| \| 2-hydroxylaurate \| Fatty Acid, Monohydroxy \| -0.06 \| -0.06 \| \| 1-(1-enyl-palmitoyl)-2-palmitoleoyl-GPC (P-16:0/16:1)* \| Plasmalogen \| -0.06 \| 0.01 \| \| linoleoyl-linoleoyl-glycerol (18:2/18:2) [1]* \| Diacylglycerol \| -0.06 \| 0.03 \| \| taurolithocholate 3-sulfate \| Secondary Bile Acid Metabolism \| -0.06 \| 0.05 \| \| 2-hydroxyphenylacetate \| Phenylalanine and Tyrosine Metabolism \| -0.06 \| 0.38 \| \| cis-4-decenoylcarnitine (C10:1) \| Fatty Acid Metabolism(Acyl Carnitine) \| -0.06 \| 0.22 \| \| palmitoyl-linoleoyl-glycerol (16:0/18:2) [2]* \| Diacylglycerol \| -0.06 \| 0.53 \| \| sphingomyelin (d18:1/20:0, d16:1/22:0)* \| Sphingolipid Metabolism \| -0.06 \| 0.33 \| \| lactosyl-N-palmitoyl-sphingosine (d18:1/16:0) \| Sphingolipid Metabolism \| -0.06 \| 0.05 \| \| dehydroisoandrosterone sulfate (DHEA-S) \| Steroid \| -0.06 \| -0.29 \| \| ethylmalonate \| Leucine, Isoleucine and Valine Metabolism \| -0.06 \| 0.18 \| \| 2-aminobutyrate \| Methionine, Cysteine, SAM and Taurine Metabolism \| -0.05 \| 0.33 \| \| phytanate \| Food Component/Plant \| -0.05 \| 0.03 \| \| 1-palmitoyl-2-oleoyl-GPE (16:0/18:1) \| Phospholipid Metabolism \| -0.05 \| 0.41 \| \| ursodeoxycholate \| Secondary Bile Acid Metabolism \| -0.05 \| 0.21 \| \| eicosapentaenoate (EPA; 20:5n3) \| Polyunsaturated Fatty Acid (n3 and n6) \| -0.05 \| 0.19 \| \| serotonin \| Tryptophan Metabolism \| -0.05 \| -0.17 \| \| theanine \| Food Component/Plant \| -0.05 \| 0.54 \| \| 1-stearoyl-2-linoleoyl-GPE (18:0/18:2)* \| Phospholipid Metabolism \| -0.05 \| 0.43 \| \| 10-undecenoate (11:1n1) \| Medium Chain Fatty Acid \| -0.05 \| 0.08 \| \| palmitate (16:0) \| Long Chain Fatty Acid \| -0.05 \| 0.23 \| \| 1-(1-enyl-stearoyl)-2-linoleoyl-GPE (P-18:0/18:2)* \| Plasmalogen \| -0.04 \| 0.51 \| \| 3-hydroxyoctanoate \| Fatty Acid, Monohydroxy \| -0.04 \| 0.59 \| \| margarate (17:0) \| Long Chain Fatty Acid \| -0.04 \| 0.28 \| \| hexanoylcarnitine (C6) \| Fatty Acid Metabolism(Acyl Carnitine) \| -0.04 \| 0.51 \| \| 1-oleoyl-GPC (18:1) \| Lysolipid \| -0.04 \| -0.09 \| \| 10-heptadecenoate (17:1n7) \| Long Chain Fatty Acid \| -0.04 \| 0.25 \| \| sphinganine \| Sphingolipid Metabolism \| -0.04 \| -0.19 \| \| linolenoylcarnitine (C18:3)* \| Fatty Acid Metabolism(Acyl Carnitine) \| -0.04 \| -0.08 \| \| vanillylmandelate (VMA) \| Phenylalanine and Tyrosine Metabolism \| -0.04 \| 0.04 \| \| isoleucine \| Leucine, Isoleucine and Valine Metabolism \| -0.04 \| -0.17 \| \| gamma-glutamylglycine \| Gamma-glutamyl Amino Acid \| -0.03 \| -0.30 \| \| palmitoyl-docosahexaenoyl-glycerol (16:0/22:6) [1]* \| Diacylglycerol \| -0.03 \| 0.38 \| \| beta-cryptoxanthin \| Food Component/Plant \| -0.03 \| 0.15 \| \| dodecanedioate \| Fatty Acid, Dicarboxylate \| -0.03 \| 0.07 \| \| sphingomyelin (d18:1/21:0, d17:1/22:0, d16:1/23:0)* \| Sphingolipid Metabolism \| -0.02 \| 0.37 \| \| 2,3-dihydroxyisovalerate \| Food Component/Plant \| -0.02 \| -0.22 \| \| 4-imidazoleacetate \| Histidine Metabolism \| -0.02 \| -0.04 \| \| oleoyl-linoleoyl-glycerol (18:1/18:2) [2] \| Diacylglycerol \| -0.02 \| 0.30 \| \| palmitoleoyl-arachidonoyl-glycerol (16:1/20:4) [2]* \| Diacylglycerol \| -0.02 \| 0.13 \| \| arginine \| Urea cycle; Arginine and Proline Metabolism \| -0.02 \| 0.11 \| \| 2-linoleoylglycerol (18:2) \| Monoacylglycerol \| -0.02 \| -0.53 \| \| sphingomyelin (d18:2/24:1, d18:1/24:2)* \| Sphingolipid Metabolism \| -0.02 \| -0.14 \| \| palmitoyl-linoleoyl-glycerol (16:0/18:2) [1]* \| Diacylglycerol \| -0.02 \| 0.47 \| \| pregnenolone sulfate \| Steroid \| -0.02 \| -0.27 \| \| 1-palmitoyl-2-alpha-linolenoyl-GPC (16:0/18:3n3)* \| Phospholipid Metabolism \| -0.02 \| 0.19 \| \| 3-hydroxybutyrylcarnitine (1) \| Fatty Acid Metabolism(Acyl Carnitine) \| -0.02 \| 0.52 \| \| 1-(1-enyl-stearoyl)-2-oleoyl-GPE (P-18:0/18:1) \| Plasmalogen \| -0.01 \| 0.51 \| \| phenylpyruvate \| Phenylalanine and Tyrosine Metabolism \| -0.01 \| -0.24 \| \| 3-methylhistidine \| Histidine Metabolism \| -0.01 \| 0.20 \| \| stearoyl-arachidonoyl-glycerol (18:0/20:4) [1]* \| Diacylglycerol \| -0.01 \| 0.50 \| \| caprate (10:0) \| Medium Chain Fatty Acid \| -0.01 \| 0.49 \| \| oleoyl-linoleoyl-glycerol (18:1/18:2) [1] \| Diacylglycerol \| -0.01 \| 0.28 \| \| N-acetylkynurenine (2) \| Tryptophan Metabolism \| -0.01 \| 0.09 \| \| 1-palmitoyl-2-arachidonoyl-GPC (16:0/20:4n6) \| Phospholipid Metabolism \| -0.01 \| 0.02 \| \| palmitoyl-arachidonoyl-glycerol (16:0/20:4) [1]* \| Diacylglycerol \| 0.00 \| 0.48 \| \| 21-hydroxypregnenolone disulfate \| Steroid \| 0.00 \| -0.20 \| \| umbelliferone sulfate \| Food Component/Plant \| 0.00 \| 0.01 \| |
| --- | --- | --- | --- | --- | --- | --- | --- | --- | --- | --- | --- | --- | --- | --- | --- | --- | --- | --- | --- | --- | --- | --- | --- | --- | --- | --- | --- | --- | --- | --- | --- | --- | --- | --- | --- | --- | --- | --- | --- | --- | --- | --- | --- | --- | --- | --- | --- | --- | --- | --- | --- | --- | --- | --- | --- | --- | --- | --- | --- | --- | --- | --- | --- | --- | --- | --- | --- | --- | --- | --- | --- | --- | --- | --- | --- | --- | --- | --- | --- | --- | --- | --- | --- | --- | --- | --- | --- | --- | --- | --- | --- | --- | --- | --- | --- | --- | --- | --- | --- | --- | --- | --- | --- | --- | --- | --- | --- | --- | --- | --- | --- | --- | --- | --- | --- | --- | --- | --- | --- | --- | --- | --- | --- | --- | --- | --- | --- | --- | --- | --- | --- | --- | --- | --- | --- | --- | --- | --- | --- | --- | --- | --- | --- | --- | --- | --- | --- | --- | --- | --- | --- | --- | --- | --- | --- | --- | --- | --- | --- | --- | --- | --- | --- | --- | --- | --- | --- | --- | --- | --- | --- | --- | --- | --- | --- | --- | --- | --- | --- | --- | --- | --- | --- | --- | --- | --- | --- | --- | --- | --- | --- | --- | --- | --- | --- | --- | --- | --- | --- | --- | --- | --- | --- | --- | --- | --- | --- | --- | --- | --- | --- | --- | --- | --- | --- | --- | --- | --- | --- | --- | --- | --- | --- | --- | --- | --- | --- | --- | --- | --- | --- | --- | --- | --- | --- | --- | --- | --- | --- | --- | --- | --- | --- | --- | --- | --- | --- | --- | --- | --- | --- | --- | --- | --- | --- | --- | --- | --- | --- | --- | --- | --- | --- | --- | --- | --- | --- | --- | --- | --- | --- | --- | --- | --- | --- | --- | --- | --- | --- | --- | --- | --- | --- | --- | --- | --- | --- | --- | --- | --- | --- | --- | --- | --- | --- | --- | --- | --- | --- | --- | --- | --- | --- | --- | --- | --- | --- | --- | --- | --- | --- | --- | --- | --- | --- | --- | --- | --- | --- | --- | --- | --- | --- | --- | --- | --- | --- | --- | --- | --- | --- | --- | --- | --- | --- | --- | --- | --- | --- | --- | --- | --- | --- | --- | --- | --- | --- | --- | --- | --- | --- | --- | --- | --- | --- | --- | --- | --- | --- | --- | --- | --- | --- | --- | --- | --- | --- | --- | --- | --- | --- | --- | --- | --- | --- | --- | --- | --- | --- | --- | --- | --- | --- | --- | --- | --- | --- | --- | --- | --- | --- | --- | --- | --- | --- | --- | --- | --- | --- | --- | --- | --- | --- | --- | --- | --- | --- | --- | --- | --- | --- | --- | --- | --- | --- | --- | --- | --- | --- | --- | --- | --- | --- | --- | --- | --- | --- | --- | --- | --- | --- | --- | --- | --- | --- | --- | --- | --- | --- | --- | --- | --- | --- | --- | --- | --- | --- | --- | --- | --- | --- | --- | --- | --- | --- | --- | --- | --- | --- | --- | --- | --- | --- | --- | --- | --- | --- | --- | --- | --- | --- | --- | --- | --- | --- | --- | --- | --- | --- | --- | --- | --- | --- | --- | --- | --- | --- | --- | --- | --- | --- | --- | --- | --- | --- | --- | --- | --- | --- | --- | --- | --- | --- | --- | --- | --- | --- | --- | --- | --- | --- | --- | --- | --- | --- | --- | --- | --- | --- | --- | --- | --- | --- | --- | --- | --- | --- | --- | --- | --- | --- | --- | --- | --- | --- | --- | --- | --- | --- | --- | --- | --- | --- | --- | --- | --- | --- | --- | --- | --- | --- | --- | --- | --- | --- | --- | --- | --- | --- | --- | --- | --- | --- | --- | --- | --- | --- | --- | --- | --- | --- | --- | --- | --- | --- | --- | --- | --- | --- | --- | --- | --- | --- | --- | --- | --- | --- | --- | --- | --- | --- | --- | --- | --- | --- | --- | --- | --- | --- | --- | --- | --- | --- | --- | --- | --- | --- | --- | --- | --- | --- | --- | --- | --- | --- | --- | --- | --- | --- | --- | --- | --- | --- | --- | --- | --- | --- | --- | --- | --- | --- | --- | --- | --- | --- | --- | --- | --- | --- | --- | --- | --- | --- | --- | --- | --- | --- | --- | --- | --- | --- | --- | --- | --- | --- | --- | --- | --- | --- | --- | --- | --- | --- | --- | --- | --- | --- | --- | --- | --- | --- | --- | --- | --- | --- | --- | --- | --- | --- | --- | --- | --- | --- | --- | --- | --- | --- | --- | --- | --- | --- | --- | --- | --- | --- | --- | --- | --- | --- | --- | --- | --- | --- | --- | --- | --- | --- | --- | --- | --- | --- | --- | --- | --- | --- | --- | --- | --- | --- | --- | --- | --- | --- | --- | --- | --- | --- | --- | --- | --- | --- | --- | --- | --- | --- | --- | --- | --- | --- | --- | --- | --- | --- | --- | --- | --- | --- | --- | --- | --- | --- | --- | --- | --- | --- | --- | --- | --- | --- | --- | --- | --- | --- | --- | --- | --- | --- | --- | --- | --- | --- | --- | --- | --- | --- | --- | --- | --- | --- | --- | --- | --- | --- | --- | --- | --- | --- | --- | --- | --- | --- | --- | --- | --- | --- | --- | --- | --- | --- | --- | --- | --- | --- | --- | --- | --- | --- | --- | --- | --- | --- | --- | --- | --- | --- | --- | --- | --- | --- | --- | --- | --- | --- | --- | --- | --- | --- | --- | --- | --- | --- | --- | --- | --- | --- | --- | --- | --- | --- | --- | --- | --- | --- | --- | --- | --- | --- | --- | --- | --- | --- | --- | --- | --- | --- | --- | --- | --- | --- | --- | --- | --- | --- | --- | --- | --- | --- | --- | --- | --- | --- | --- | --- | --- | --- | --- | --- | --- | --- | --- | --- | --- | --- | --- | --- | --- | --- | --- | --- | --- | --- | --- | --- | --- | --- | --- | --- | --- | --- | --- | --- | --- | --- | --- | --- | --- | --- | --- | --- | --- | --- | --- | --- | --- | --- | --- | --- | --- | --- | --- | --- | --- | --- | --- | --- | --- | --- | --- | --- | --- | --- | --- | --- | --- | --- | --- | --- | --- | --- | --- | --- | --- | --- | --- | --- | --- | --- | --- | --- | --- | --- | --- | --- | --- | --- | --- | --- | --- | --- | --- | --- | --- | --- | --- | --- | --- | --- | --- | --- | --- | --- | --- | --- | --- | --- | --- | --- | --- | --- | --- | --- | --- | --- | --- | --- | --- | --- | --- | --- | --- | --- | --- | --- | --- | --- | --- | --- | --- | --- | --- | --- | --- | --- | --- | --- | --- | --- | --- | --- | --- | --- | --- | --- | --- | --- | --- | --- | --- | --- | --- | --- | --- | --- | --- | --- | --- | --- | --- | --- | --- | --- | --- | --- | --- | --- | --- | --- | --- | --- | --- | --- | --- | --- | --- | --- | --- | --- | --- | --- | --- | --- | --- | --- | --- | --- | --- | --- | --- | --- | --- | --- | --- | --- | --- | --- | --- | --- | --- | --- | --- | --- | --- | --- | --- | --- | --- | --- | --- | --- | --- | --- | --- | --- | --- | --- | --- | --- | --- | --- | --- | --- | --- | --- | --- | --- | --- | --- | --- | --- | --- | --- | --- | --- | --- | --- | --- | --- | --- | --- | --- | --- | --- | --- | --- | --- | --- | --- | --- | --- | --- | --- | --- | --- | --- | --- | --- | --- | --- | --- | --- | --- | --- | --- | --- | --- | --- | --- | --- | --- | --- | --- | --- | --- | --- | --- | --- | --- | --- | --- | --- | --- | --- | --- | --- | --- | --- | --- | --- | --- | --- | --- | --- | --- | --- | --- | --- | --- | --- | --- | --- | --- | --- | --- | --- | --- | --- | --- | --- | --- | --- | --- | --- | --- | --- | --- | --- | --- | --- | --- | --- | --- | --- | --- | --- | --- | --- | --- | --- | --- | --- | --- | --- | --- | --- | --- | --- | --- | --- | --- | --- | --- | --- | --- | --- | --- | --- | --- | --- | --- | --- | --- | --- | --- | --- | --- | --- | --- | --- | --- | --- | --- | --- | --- | --- | --- | --- | --- | --- | --- | --- | --- | --- | --- | --- | --- | --- | --- | --- | --- | --- | --- | --- | --- | --- | --- | --- | --- | --- | --- | --- | --- | --- | --- | --- | --- | --- | --- | --- | --- | --- | --- | --- | --- | --- | --- | --- | --- | --- | --- | --- | --- | --- | --- | --- | --- | --- | --- | --- | --- | --- | --- | --- | --- | --- | --- | --- | --- | --- | --- | --- | --- | --- | --- | --- | --- | --- | --- | --- | --- | --- | --- | --- | --- | --- | --- | --- | --- | --- | --- | --- | --- | --- | --- | --- | --- | --- | --- | --- | --- | --- | --- | --- | --- | --- | --- | --- | --- | --- | --- | --- | --- | --- | --- | --- | --- | --- | --- | --- | --- | --- | --- | --- | --- | --- | --- | --- | --- | --- | --- | --- | --- | --- | --- | --- | --- | --- | --- | --- | --- | --- | --- | --- | --- | --- | --- | --- | --- | --- | --- | --- | --- | --- | --- | --- | --- | --- | --- | --- | --- | --- | --- | --- | --- | --- | --- | --- | --- | --- | --- | --- | --- | --- | --- | --- | --- | --- | --- | --- | --- | --- | --- | --- | --- | --- | --- | --- | --- | --- | --- | --- | --- | --- | --- | --- | --- | --- | --- | --- | --- | --- | --- | --- | --- | --- | --- | --- | --- | --- | --- | --- | --- | --- | --- | --- | --- | --- | --- | --- | --- | --- | --- | --- | --- | --- | --- | --- | --- | --- | --- | --- | --- | --- | --- | --- | --- | --- | --- | --- | --- | --- | --- | --- | --- | --- | --- | --- | --- | --- | --- | --- | --- | --- | --- | --- | --- | --- | --- | --- | --- | --- | --- | --- | --- | --- | --- | --- | --- | --- | --- | --- | --- | --- | --- | --- | --- | --- | --- | --- | --- | --- | --- | --- | --- | --- | --- | --- | --- | --- | --- | --- | --- | --- | --- | --- | --- | --- | --- | --- | --- | --- | --- | --- | --- | --- | --- | --- | --- | --- | --- | --- | --- | --- | --- | --- | --- | --- | --- | --- | --- | --- | --- | --- | --- | --- | --- | --- | --- | --- | --- | --- | --- | --- | --- | --- | --- | --- | --- | --- | --- | --- | --- | --- | --- | --- | --- | --- | --- | --- | --- | --- | --- | --- | --- | --- | --- | --- | --- | --- | --- | --- | --- | --- | --- | --- | --- | --- | --- | --- | --- | --- | --- | --- | --- | --- | --- | --- | --- | --- | --- | --- | --- | --- | --- | --- | --- | --- | --- | --- | --- | --- | --- | --- | --- | --- | --- | --- | --- | --- | --- | --- | --- | --- | --- | --- | --- | --- | --- | --- | --- | --- | --- | --- | --- | --- | --- | --- | --- | --- | --- | --- | --- | --- | --- | --- | --- | --- | --- | --- | --- | --- | --- | --- | --- | --- | --- | --- | --- | --- | --- | --- | --- | --- | --- | --- | --- | --- | --- | --- | --- | --- | --- | --- | --- | --- | --- | --- | --- | --- | --- | --- | --- | --- | --- | --- | --- | --- | --- | --- | --- | --- | --- | --- | --- | --- | --- | --- | --- | --- | --- | --- | --- | --- | --- | --- | --- | --- | --- | --- | --- | --- | --- | --- | --- | --- | --- | --- | --- | --- | --- | --- | --- | --- | --- | --- | --- | --- | --- | --- | --- | --- | --- | --- | --- | --- | --- | --- | --- | --- | --- | --- | --- | --- | --- | --- | --- | --- | --- | --- | --- | --- | --- | --- | --- | --- | --- | --- | --- | --- | --- | --- | --- | --- | --- | --- | --- | --- | --- | --- | --- | --- | --- | --- | --- | --- | --- | --- | --- | --- | --- | --- | --- | --- | --- | --- | --- | --- | --- | --- | --- | --- | --- | --- | --- | --- | --- | --- | --- | --- | --- | --- | --- | --- | --- | --- | --- | --- | --- | --- | --- | --- | --- | --- | --- | --- | --- | --- | --- | --- | --- | --- | --- | --- | --- | --- | --- | --- | --- | --- | --- | --- | --- | --- | --- | --- | --- | --- | --- | --- | --- | --- | --- | --- | --- | --- | --- | --- | --- | --- | --- | --- | --- | --- | --- | --- | --- | --- | --- | --- | --- | --- | --- | --- | --- | --- | --- | --- | --- | --- | --- | --- | --- | --- | --- | --- | --- | --- | --- | --- | --- | --- | --- | --- | --- | --- | --- | --- | --- | --- | --- | --- | --- | --- | --- | --- | --- | --- | --- | --- | --- | --- | --- | --- | --- | --- | --- | --- |

**Supplementary Table S5**. Pearson correlation coefficient between IL-1ra and all measured metabolites in the Null condition for both LTBI and TB.

| TNFa signature |
| --- |
| C3 |
| CCL4 |
| CD44 |
| CD83 |
| IRAK2 |
| NFKB1 |
| NFKB2 |
| NFKBIA |
| NFKBIZ |
| POU2F2 |
| RELB |
| SOCS3 |
| SRC |
| TNFAIP3 |

**Supplementary Table S6.** Unique cytokine-induced gene signature derived from TNF single cytokine stimulation as previously described^15^.

| \| **Gene** \| **p-value** \| **q-value** \| **Fold change** \| \| --- \| --- \| --- \| --- \| \| IL1A \| 3.43E-31 \| 2.09E-28 \| 212.311701 \| \| CCL20 \| 3.54E-29 \| 7.20E-27 \| 300.568543 \| \| IL6 \| 2.95E-29 \| 7.20E-27 \| 136.070461 \| \| IL1B \| 1.39E-28 \| 2.13E-26 \| 84.2357019 \| \| CCL19 \| 4.53E-25 \| 5.52E-23 \| 23.7958806 \| \| IL10 \| 2.52E-24 \| 2.56E-22 \| 22.4348548 \| \| CCL2 \| 4.46E-22 \| 3.89E-20 \| 69.4070409 \| \| CXCL2 \| 6.80E-21 \| 5.19E-19 \| 17.6122855 \| \| CXCL1 \| 5.58E-20 \| 3.78E-18 \| 7.24472043 \| \| C3 \| 2.52E-19 \| 1.54E-17 \| 25.3160454 \| \| CCL3 \| 3.31E-19 \| 1.84E-17 \| 12.3458144 \| \| BATF3 \| 7.24E-18 \| 3.68E-16 \| 10.3338066 \| \| CCL4 \| 8.44E-18 \| 3.96E-16 \| 5.72422376 \| \| LILRB1 \| 1.10E-16 \| 4.80E-15 \| 3.90816135 \| \| SOCS3 \| 9.14E-16 \| 3.72E-14 \| 5.15177685 \| \| BATF \| 2.88E-15 \| 1.10E-13 \| 3.41543669 \| \| ADA \| 5.79E-14 \| 1.96E-12 \| 2.7834998 \| \| CD80 \| 5.78E-14 \| 1.96E-12 \| 4.43455667 \| \| TNFSF15 \| 1.98E-13 \| 6.36E-12 \| 16.4780084 \| \| SELPLG \| 2.90E-13 \| 8.86E-12 \| 0.37229031 \| \| IRAK2 \| 5.89E-13 \| 1.71E-11 \| 6.46316131 \| \| LAMP3 \| 8.68E-13 \| 2.41E-11 \| 5.92884047 \| \| PTGS2 \| 1.04E-12 \| 2.76E-11 \| 14.6649475 \| \| LAIR1 \| 2.07E-12 \| 5.25E-11 \| 2.13989502 \| \| POU2F2 \| 3.79E-12 \| 9.24E-11 \| 2.26164102 \| \| CD1D \| 1.04E-11 \| 2.45E-10 \| 0.2482714 \| \| TNFAIP6 \| 1.12E-11 \| 2.53E-10 \| 4.95347156 \| \| CCL22 \| 1.36E-11 \| 2.96E-10 \| 7.0972002 \| \| NFKBIZ \| 1.47E-11 \| 3.10E-10 \| 3.13342401 \| \| NFKB1 \| 1.78E-11 \| 3.61E-10 \| 3.07008809 \| \| EBI3 \| 2.23E-11 \| 4.39E-10 \| 2.84525892 \| \| LILRB4 \| 2.58E-11 \| 4.91E-10 \| 4.50950805 \| \| TNFRSF9 \| 4.24E-11 \| 7.84E-10 \| 4.03643003 \| \| SRC \| 8.78E-11 \| 1.57E-09 \| 4.238783 \| \| MSR1 \| 9.04E-11 \| 1.58E-09 \| 0.24770417 \| \| LILRA3 \| 2.29E-10 \| 3.87E-09 \| 2.91298853 \| \| SLAMF7 \| 4.28E-10 \| 7.05E-09 \| 2.76580607 \| \| CD40LG \| 1.66E-09 \| 2.66E-08 \| 0.57192069 \| \| TNF \| 4.27E-09 \| 6.69E-08 \| 2.29017836 \| \| FCER1A \| 5.09E-09 \| 7.76E-08 \| 0.23207131 \| \| CD14 \| 5.23E-09 \| 7.77E-08 \| 2.14369557 \| \| CLEC5A \| 6.73E-09 \| 9.55E-08 \| 4.81805219 \| \| TRAF1 \| 6.62E-09 \| 9.55E-08 \| 2.03390181 \| \| CCR2 \| 7.78E-09 \| 1.08E-07 \| 0.33110713 \| \| IL2RA \| 7.98E-09 \| 1.08E-07 \| 4.1747798 \| \| CCL7 \| 1.22E-08 \| 1.62E-07 \| 5.64608199 \| \| DUSP4 \| 1.39E-08 \| 1.81E-07 \| 4.29974158 \| \| IL23A \| 2.00E-08 \| 2.54E-07 \| 4.27694314 \| \| NFKBIA \| 2.07E-08 \| 2.58E-07 \| 2.57754936 \| \| IL8 \| 2.14E-08 \| 2.62E-07 \| 3.44525325 \| \| AHR \| 2.74E-08 \| 3.27E-07 \| 1.55085627 \| \| ALDH1A1 \| 5.11E-08 \| 5.99E-07 \| 0.31581903 \| \| ICAM5 \| 9.11E-08 \| 1.05E-06 \| 3.73567785 \| \| CXCL13 \| 1.22E-07 \| 1.35E-06 \| 2.24874481 \| \| IRAK3 \| 1.22E-07 \| 1.35E-06 \| 2.88747799 \| \| CD274 \| 1.64E-07 \| 1.79E-06 \| 6.21347375 \| \| CDKN1A \| 1.67E-07 \| 1.79E-06 \| 3.60580151 \| \| PDCD1LG2 \| 4.50E-07 \| 4.74E-06 \| 3.24891949 \| \| KCNJ2 \| 7.52E-07 \| 7.78E-06 \| 3.48865275 \| \| CASP8 \| 9.28E-07 \| 9.44E-06 \| 0.60129254 \| \| PLAU \| 1.04E-06 \| 1.04E-05 \| 7.67171924 \| \| RELB \| 1.72E-06 \| 1.70E-05 \| 2.1874802 \| \| CCRL2 \| 1.86E-06 \| 1.78E-05 \| 3.64517996 \| \| CD40 \| 1.87E-06 \| 1.78E-05 \| 2.21657161 \| \| TNFAIP3 \| 2.13E-06 \| 2.00E-05 \| 2.01128837 \| \| TNFRSF4 \| 2.23E-06 \| 2.06E-05 \| 2.53379536 \| \| TNFSF12 \| 3.45E-06 \| 3.14E-05 \| 0.82198616 \| \| CASP2 \| 3.94E-06 \| 3.53E-05 \| 0.77089325 \| \| CD46 \| 4.33E-06 \| 3.82E-05 \| 0.60245566 \| \| IL7R \| 5.78E-06 \| 4.96E-05 \| 0.72868945 \| \| NFATC3 \| 5.78E-06 \| 4.96E-05 \| 0.72909413 \| \| HLA.DMB \| 5.86E-06 \| 4.97E-05 \| 0.72039147 \| \| ICAM1 \| 6.12E-06 \| 5.11E-05 \| 2.35325886 \| \| JAK3 \| 6.48E-06 \| 5.34E-05 \| 1.63910012 \| \| IL16 \| 6.67E-06 \| 5.42E-05 \| 0.68323606 \| \| MARCO \| 7.47E-06 \| 6.00E-05 \| 5.50605871 \| \| CSF3R \| 7.69E-06 \| 6.09E-05 \| 0.51794721 \| \| SELL \| 8.03E-06 \| 6.28E-05 \| 0.6690134 \| \| PYCARD \| 9.99E-06 \| 7.72E-05 \| 0.5088453 \| \| BID \| 1.18E-05 \| 8.99E-05 \| 2.97096169 \| \| S1PR1 \| 1.59E-05 \| 0.00011975 \| 0.69552438 \| \| ITGA4 \| 1.81E-05 \| 0.00013479 \| 0.76409875 \| \| IDO1 \| 1.85E-05 \| 0.00013584 \| 2.51373061 \| \| CXCR2 \| 2.25E-05 \| 0.00016354 \| 0.52178957 \| \| LILRA1 \| 2.45E-05 \| 0.00017617 \| 2.45722501 \| \| IKBKE \| 2.63E-05 \| 0.0001865 \| 1.53738768 \| \| CYB561 \| 3.04E-05 \| 0.00021345 \| 0.66560952 \| \| KLF2 \| 3.60E-05 \| 0.00024923 \| 0.66820886 \| \| TICAM1 \| 4.13E-05 \| 0.00028295 \| 1.97800218 \| \| CCL23 \| 4.35E-05 \| 0.00029163 \| 2.11730627 \| \| CYBB \| 4.35E-05 \| 0.00029163 \| 1.4926294 \| \| IL1RN \| 4.83E-05 \| 0.00031993 \| 3.74745204 \| \| NFKB2 \| 4.89E-05 \| 0.00032091 \| 1.91395377 \| \| CD36 \| 5.50E-05 \| 0.00035679 \| 0.58312416 \| \| ATG7 \| 5.86E-05 \| 0.00037657 \| 1.87104598 \| \| SELE \| 6.10E-05 \| 0.00038737 \| 1.53308961 \| \| FER1L3 \| 6.28E-05 \| 0.00039472 \| 2.6818528 \| \| CTSS \| 7.77E-05 \| 0.0004836 \| 0.64444716 \| \| CD83 \| 8.07E-05 \| 0.0004923 \| 1.86846557 \| \| IFIT2 \| 8.07E-05 \| 0.0004923 \| 0.45531283 \| \| CISH \| 9.29E-05 \| 0.00056106 \| 2.81329332 \| \| GAS6 \| 9.50E-05 \| 0.00056824 \| 1.47604225 \| \| CD244 \| 0.00010707 \| 0.0006341 \| 0.72778338 \| \| BCL3 \| 0.00012504 \| 0.00073339 \| 2.07021415 \| \| HLA.DMA \| 0.00012737 \| 0.00073998 \| 0.72211274 \| \| CCND3 \| 0.00014055 \| 0.00080884 \| 0.77631712 \| \| SIGIRR \| 0.00017294 \| 0.00098592 \| 0.78599771 \| \| CASP10 \| 0.00019282 \| 0.00108908 \| 1.85097323 \| \| LTB4R2 \| 0.00019467 \| 0.00108943 \| 0.63438784 \| \| CLEC7A \| 0.00022668 \| 0.00125702 \| 0.6194544 \| \| TNFSF10 \| 0.00029406 \| 0.00161602 \| 0.61687154 \| \| CCL18 \| 0.00029984 \| 0.00163304 \| 1.43183676 \| \| PTAFR \| 0.00031731 \| 0.00171291 \| 1.62913479 \| \| CD82 \| 0.00036271 \| 0.00194083 \| 1.80731 \| \| IL23R \| 0.00036593 \| 0.00194101 \| 2.08019679 \| \| CFD \| 0.00039919 \| 0.00209919 \| 0.41915334 \| \| ITGAL \| 0.00051134 \| 0.00266596 \| 0.71677539 \| \| CD96 \| 0.00061903 \| 0.00320007 \| 0.78448023 \| \| ICOSLG \| 0.00065448 \| 0.00335488 \| 1.8685809 \| \| SPP1 \| 0.00066704 \| 0.00339079 \| 5.24273659 \| \| CARD9 \| 0.00071088 \| 0.00358379 \| 1.89067577 \| \| CXCL10 \| 0.00071791 \| 0.00358957 \| 2.13308281 \| \| HPSE \| 0.00073118 \| 0.00362619 \| 2.09786235 \| \| HAVCR2 \| 0.00075553 \| 0.00367935 \| 1.96191698 \| \| TGFBR2 \| 0.00075583 \| 0.00367935 \| 0.72573864 \| \| VAMP5 \| 0.00076 \| 0.00367935 \| 0.75889806 \| \| LCK \| 0.00079416 \| 0.00381445 \| 0.87109985 \| \| CD27 \| 0.00085273 \| 0.00406377 \| 0.75457751 \| \| FCGRT \| 0.00089073 \| 0.00421198 \| 0.73925756 \| \| SOCS1 \| 0.0009399 \| 0.00441031 \| 2.00084581 \| \| CD3E \| 0.00129682 \| 0.00603863 \| 0.77590935 \| \| LAG3 \| 0.00140841 \| 0.00650856 \| 1.90832121 \| \| PECAM1 \| 0.00150262 \| 0.00689172 \| 0.71562813 \| \| LHFPL2 \| 0.00152647 \| 0.00694886 \| 2.37025136 \| \| KLRK1 \| 0.0015997 \| 0.00722827 \| 0.81133409 \| \| CD22 \| 0.00177202 \| 0.00794803 \| 2.39445935 \| \| ICAM3 \| 0.0019465 \| 0.0086669 \| 0.74639282 \| \| IL1R2 \| 0.00200718 \| 0.00887232 \| 1.76261942 \| \| CSF2 \| 0.00229442 \| 0.01006904 \| 1.22930763 \| \| CCL5 \| 0.0026367 \| 0.01148848 \| 0.74536035 \| \| CLEC6A \| 0.00274293 \| 0.0118462 \| 2.10609416 \| \| KLRB1 \| 0.00275764 \| 0.0118462 \| 0.78962194 \| \| IGF2R \| 0.00297059 \| 0.01267175 \| 0.75002209 \| \| CD4 \| 0.00300011 \| 0.0127088 \| 0.82125491 \| \| ABCB1 \| 0.00307494 \| 0.01293595 \| 0.8313858 \| \| POLR1B \| 0.00319791 \| 0.01336113 \| 1.8567145 \| \| IL1R1 \| 0.00324724 \| 0.01341943 \| 2.66620929 \| \| TLR2 \| 0.00327786 \| 0.01341943 \| 1.55679825 \| \| sCTLA4 \| 0.00327617 \| 0.01341943 \| 1.97778007 \| \| CD163 \| 0.00335888 \| 0.01365945 \| 1.57298181 \| \| TLR9 \| 0.00363583 \| 0.01468779 \| 0.56050961 \| \| IRF8 \| 0.00368463 \| 0.014787 \| 0.7829239 \| \| FCAR \| 0.00385377 \| 0.0153647 \| 1.38541194 \| \| C1QB \| 0.00416485 \| 0.01628563 \| 1.7231377 \| \| IFNA1.13 \| 0.00415787 \| 0.01628563 \| 1.22245725 \| \| MBP \| 0.0041368 \| 0.01628563 \| 0.79074532 \| \| CD247 \| 0.00429836 \| 0.01659493 \| 0.85589517 \| \| ICAM2 \| 0.00427783 \| 0.01659493 \| 0.89108688 \| \| STAT6 \| 0.00442326 \| 0.01696974 \| 0.70405303 \| \| ETS1 \| 0.00461676 \| 0.0176014 \| 0.82120368 \| \| TRAF3 \| 0.00468297 \| 0.01774293 \| 1.44733713 \| \| ZAP70 \| 0.00475 \| 0.0178858 \| 0.81264434 \| \| GZMA \| 0.00484075 \| 0.01800523 \| 0.75953587 \| \| PSMB10 \| 0.00481794 \| 0.01800523 \| 0.84883497 \| \| TCF7 \| 0.00524422 \| 0.01938772 \| 0.80390393 \| \| IFNG \| 0.00571594 \| 0.02075431 \| 1.21169367 \| \| NLRP3 \| 0.00565143 \| 0.02075431 \| 1.64264762 \| \| TNFSF8 \| 0.00570429 \| 0.02075431 \| 1.46439223 \| \| CD5 \| 0.00575757 \| 0.02075554 \| 0.82957309 \| \| ITGA6 \| 0.00578433 \| 0.02075554 \| 1.18908924 \| \| FYN \| 0.00603091 \| 0.02151377 \| 0.7897243 \| \| MAP4K1 \| 0.00640358 \| 0.02244933 \| 0.85128965 \| \| MAPK11 \| 0.00638657 \| 0.02244933 \| 0.57492036 \| \| SH2D1A \| 0.00634492 \| 0.02244933 \| 0.82758476 \| \| TBX21 \| 0.00649259 \| 0.02263131 \| 0.81782609 \| \| FCGR3A.B \| 0.00655248 \| 0.0227103 \| 0.65242591 \| \| PLAUR \| 0.00673515 \| 0.02321153 \| 1.90470689 \| \| IFNAR2 \| 0.00679705 \| 0.02329326 \| 0.73547948 \| \| TRAF2 \| 0.00737003 \| 0.02511574 \| 1.71960001 \| \| CXCR3 \| 0.0075181 \| 0.02537897 \| 0.81245398 \| \| IL17F \| 0.00753048 \| 0.02537897 \| 1.10094317 \| \| NCF4 \| 0.00771893 \| 0.02587114 \| 0.73172274 \| \| ENTPD1 \| 0.00797935 \| 0.02659783 \| 0.66945316 \| \| NOTCH1 \| 0.0080744 \| 0.02676839 \| 0.76211732 \| \| LEF1 \| 0.00879035 \| 0.0289844 \| 0.89700691 \| \| CSF2RB \| 0.00902795 \| 0.02950632 \| 1.40375874 \| \| CXCL9 \| 0.00909375 \| 0.02950632 \| 1.33990527 \| \| MAP4K2 \| 0.00906815 \| 0.02950632 \| 0.82301292 \| \| CCR7 \| 0.00931243 \| 0.03005599 \| 1.25983431 \| \| CD9 \| 0.0103004 \| 0.03306971 \| 0.58875487 \| \| GBP2 \| 0.0105318 \| 0.03363559 \| 0.69579201 \| \| IL12RB1 \| 0.011472 \| 0.0364475 \| 0.60897927 \| \| SMAD3 \| 0.0117892 \| 0.0372612 \| 1.28071991 \| \| ARHGDIB \| 0.0120456 \| 0.03787534 \| 0.85433097 \| \| TRAFD1 \| 0.0121496 \| 0.03800644 \| 0.74259937 \| \| PML \| 0.0123071 \| 0.03830271 \| 0.76551789 \| \| LTBR \| 0.012402 \| 0.03840213 \| 2.00848815 \| \| IL6R \| 0.0125199 \| 0.03857141 \| 0.75910587 \| \| CD209 \| 0.0132276 \| 0.04053603 \| 1.37714933 \| \| FCER1G \| 0.0132905 \| 0.04053603 \| 1.49507825 \| \| IL12B \| 0.0136527 \| 0.04143357 \| 1.22335744 \| \| MAPK1 \| 0.0137494 \| 0.04152047 \| 0.80608116 \| \| HLA.C \| 0.0138663 \| 0.04166721 \| 0.71163926 \| \| KREMEN1 \| 0.0142776 \| 0.04269282 \| 2.14163102 \| \| NFATC2 \| 0.014464 \| 0.04303922 \| 0.80822451 \| \| PPBP \| 0.0147094 \| 0.04355696 \| 0.75049637 \| \| CXCR1 \| 0.0154676 \| 0.04558085 \| 0.67646679 \| \| SDHA \| 0.015728 \| 0.04612538 \| 0.87478234 \| \| ARG2 \| 0.0158037 \| 0.04612563 \| 1.61150088 \| \| ABL1 \| 0.0171857 \| 0.04992037 \| 0.82545473 \| \| CD99 \| 0.0179527 \| 0.05190117 \| 0.8267941 \| \| MR1 \| 0.0181272 \| 0.05215845 \| 1.67273741 \| \| CCR1 \| 0.0182493 \| 0.05226325 \| 1.67253452 \| \| CD28 \| 0.0189324 \| 0.05371518 \| 0.86730721 \| \| NOD1 \| 0.0189039 \| 0.05371518 \| 0.71072877 \| \| TRAF4 \| 0.0194128 \| 0.05482319 \| 1.80643831 \| \| IL21R \| 0.0195709 \| 0.05501497 \| 1.58548255 \| \| BCL2L11 \| 0.0206973 \| 0.05791446 \| 0.88999183 \| \| CTLA4.TM \| 0.0214969 \| 0.05987721 \| 1.80442979 \| \| CD44 \| 0.0219813 \| 0.06091361 \| 1.29736012 \| \| FAS \| 0.0220687 \| 0.06091361 \| 0.71111214 \| \| BCL2 \| 0.0225096 \| 0.0618507 \| 0.91879697 \| \| CLEC4A \| 0.0228753 \| 0.06257369 \| 1.44631318 \| \| DEFB1 \| 0.0236658 \| 0.06416061 \| 0.82098571 \| \| TRAF5 \| 0.0236122 \| 0.06416061 \| 0.9492829 \| \| PAX5 \| 0.0245032 \| 0.0658456 \| 0.60878469 \| \| Sep-04 \| 0.024423 \| 0.0658456 \| 0.57727473 \| \| CD79B \| 0.025349 \| 0.06781969 \| 0.7502774 \| \| DEFA1 \| 0.0261553 \| 0.06967132 \| 0.68171377 \| \| ICOS \| 0.02676 \| 0.07066494 \| 1.54661702 \| \| KLRC2 \| 0.026656 \| 0.07066494 \| 0.86731502 \| \| IL32 \| 0.0280509 \| 0.07343798 \| 0.67748363 \| \| JAK2 \| 0.0280509 \| 0.07343798 \| 0.79497257 \| \| CCR10 \| 0.0285155 \| 0.07433528 \| 0.89192358 \| \| JAK1 \| 0.0289307 \| 0.0747785 \| 0.82202888 \| \| RARRES3 \| 0.0289307 \| 0.0747785 \| 0.94118589 \| \| CD3D \| 0.0298342 \| 0.07675927 \| 0.84863318 \| \| TNFRSF10C \| 0.0299487 \| 0.07675927 \| 0.75268911 \| \| BTLA \| 0.030411 \| 0.07761803 \| 0.87786804 \| \| HLA.DPB1 \| 0.031956 \| 0.0812215 \| 0.81986655 \| \| TNFRSF8 \| 0.0342048 \| 0.08657646 \| 1.70489041 \| \| LILRA2 \| 0.0349853 \| 0.08818609 \| 0.82799503 \| \| CRADD \| 0.0358408 \| 0.08997073 \| 1.62697492 \| \| PDCD2 \| 0.0361369 \| 0.08997351 \| 0.79849596 \| \| SMARCD3 \| 0.0359919 \| 0.08997351 \| 0.58801709 \| \| MS4A1 \| 0.0370005 \| 0.09174921 \| 0.83399636 \| \| XCR1 \| 0.0371854 \| 0.09183439 \| 0.81753477 \| \| CEBPB \| 0.0376939 \| 0.09271483 \| 1.36084524 \| \| CD74 \| 0.0388263 \| 0.09511664 \| 0.82851689 \| \| LTB4R \| 0.0401344 \| 0.09792794 \| 0.79819602 \| \| CREB5 \| 0.0419357 \| 0.10191545 \| 0.70124622 \| \| CD276 \| 0.0431552 \| 0.10405009 \| 1.59065727 \| \| IL28A.B \| 0.0430355 \| 0.10405009 \| 0.94951237 \| \| SLC2A1 \| 0.0448919 \| 0.10781126 \| 1.70450996 \| \| IL13RA1 \| 0.0455834 \| 0.10904264 \| 0.82272604 \| \| SMAD5 \| 0.0464061 \| 0.11057704 \| 1.05105911 \| \| CLEC4E \| 0.0472501 \| 0.11215004 \| 1.35900616 \| \| NT5E \| 0.048583 \| 0.11486678 \| 1.67988098 \| \| TLR1 \| 0.048968 \| 0.11533004 \| 0.80880735 \| \| IRF1 \| 0.0500236 \| 0.11736306 \| 0.78108095 \| \| CX3CR1 \| 0.0509164 \| 0.11900002 \| 0.79734942 \| \| CCL8 \| 0.0512057 \| 0.11921938 \| 1.76455938 \| \| KIT \| 0.0518135 \| 0.1201758 \| 0.80363649 \| \| SLAMF6 \| 0.0528695 \| 0.12216059 \| 0.81562393 \| \| BTK \| 0.0540587 \| 0.12435033 \| 0.84838205 \| \| KLRG2 \| 0.0542249 \| 0.12435033 \| 0.86862598 \| \| IRF3 \| 0.0561793 \| 0.12787079 \| 0.73040776 \| \| KLRG1 \| 0.056164 \| 0.12787079 \| 0.70173295 \| \| CD58 \| 0.057559 \| 0.13052413 \| 1.23379696 \| \| PTK2 \| 0.0583616 \| 0.13185399 \| 0.83897981 \| \| IFNAR1 \| 0.0597879 \| 0.13457793 \| 1.34815558 \| \| APOL6 \| 0.0612457 \| 0.13735249 \| 0.79885911 \| \| HLA.A \| 0.0616672 \| 0.13779118 \| 0.83238104 \| \| CD55 \| 0.0624808 \| 0.13909959 \| 0.69885206 \| \| CSF1R \| 0.0630671 \| 0.13938743 \| 1.65715666 \| \| STAT5B \| 0.062946 \| 0.13938743 \| 0.82923964 \| \| MME \| 0.0633755 \| 0.13956338 \| 0.66101871 \| \| GBP5 \| 0.0655693 \| 0.14387508 \| 0.70290566 \| \| CD24 \| 0.0669112 \| 0.1457778 \| 0.88280579 \| \| PSMB8 \| 0.0669144 \| 0.1457778 \| 0.84504379 \| \| PSMB5 \| 0.0682731 \| 0.14820851 \| 1.58611787 \| \| PSMB9 \| 0.0701414 \| 0.1517243 \| 0.81242357 \| \| TNFRSF13C \| 0.0730081 \| 0.15736728 \| 0.88096707 \| \| CAMP \| 0.073963 \| 0.15886419 \| 0.688927 \| \| STAT2 \| 0.0769787 \| 0.16476143 \| 0.86756094 \| \| CASP1 \| 0.0805956 \| 0.17070638 \| 1.23722937 \| \| TLR8 \| 0.0800706 \| 0.17070638 \| 0.75788192 \| \| TMEM173 \| 0.0805958 \| 0.17070638 \| 0.94737703 \| \| IKBKAP \| 0.0810036 \| 0.17097646 \| 0.94802598 \| \| ATM \| 0.0819438 \| 0.17236454 \| 0.71593722 \| \| GBP4 \| 0.0838049 \| 0.1756735 \| 0.80465764 \| \| CEACAM1 \| 0.0841446 \| 0.17578153 \| 1.64022532 \| \| KLRF2 \| 0.0844598 \| 0.17583781 \| 1.07387644 \| \| C4A.B \| 0.0850947 \| 0.17655703 \| 0.66902777 \| \| IL10RA \| 0.0879606 \| 0.18188463 \| 0.86882228 \| \| APOL1 \| 0.0916957 \| 0.18644833 \| 0.87767821 \| \| CD79A \| 0.0911131 \| 0.18644833 \| 0.85576525 \| \| CD81 \| 0.0916959 \| 0.18644833 \| 1.23170093 \| \| KLRC4 \| 0.0916545 \| 0.18644833 \| 0.82044073 \| \| LILRB2 \| 0.0911131 \| 0.18644833 \| 1.30401729 \| \| NCAM1 \| 0.0922502 \| 0.18695223 \| 0.75256442 \| \| ETV7 \| 0.0992554 \| 0.20048276 \| 0.63763115 \| \| CD45RB \| 0.103348 \| 0.20806033 \| 1.15826792 \| \| BCL6 \| 0.106602 \| 0.21390533 \| 1.33763732 \| \| RAF1 \| 0.109264 \| 0.21781386 \| 0.82334329 \| \| TIGIT \| 0.109264 \| 0.21781386 \| 0.89377029 \| \| ITGB2 \| 0.111978 \| 0.222497 \| 0.83965874 \| \| TGFB1 \| 0.113355 \| 0.22450179 \| 0.82002967 \| \| IL6ST \| 0.114396 \| 0.22583029 \| 1.15947925 \| \| CTNNB1 \| 0.115445 \| 0.22716597 \| 1.15382306 \| \| IL1RL1 \| 0.118917 \| 0.23259965 \| 1.51005487 \| \| TNFSF13B \| 0.118969 \| 0.23259965 \| 0.98631139 \| \| MALT1 \| 0.124109 \| 0.24187377 \| 0.91407852 \| \| PRF1 \| 0.126352 \| 0.24546089 \| 0.84738765 \| \| CD53 \| 0.128627 \| 0.24908721 \| 1.24482765 \| \| CMKLR1 \| 0.131754 \| 0.25419681 \| 0.69474287 \| \| HLA.DRA \| 0.132099 \| 0.25419681 \| 1.13061755 \| \| C14orf166 \| 0.135246 \| 0.25943415 \| 0.90161911 \| \| PSMC2 \| 0.137242 \| 0.26243768 \| 1.21821942 \| \| IL7 \| 0.139108 \| 0.26517462 \| 0.88131948 \| \| HLA.B \| 0.139667 \| 0.26541081 \| 0.84691847 \| \| AIRE \| 0.14086 \| 0.26684658 \| 0.93640828 \| \| CD6 \| 0.142125 \| 0.26758102 \| 0.90415812 \| \| MIF \| 0.142125 \| 0.26758102 \| 1.34301833 \| \| CCR5 \| 0.143762 \| 0.26941043 \| 1.23552826 \| \| LIF \| 0.14398 \| 0.26941043 \| 1.81056879 \| \| IFITM1 \| 0.148414 \| 0.27601384 \| 1.18226054 \| \| IRAK1 \| 0.147986 \| 0.27601384 \| 1.43674796 \| \| DUSP3 \| 0.150558 \| 0.27915009 \| 1.30344798 \| \| DEFB4A \| 0.156091 \| 0.28593246 \| 0.96618583 \| \| IL20 \| 0.156091 \| 0.28593246 \| 0.97400494 \| \| RAG1 \| 0.156091 \| 0.28593246 \| 0.97013566 \| \| THY1 \| 0.156091 \| 0.28593246 \| 0.97783239 \| \| AICDA \| 0.160932 \| 0.29079776 \| 1.05879288 \| \| CXCL11 \| 0.160932 \| 0.29079776 \| 1.06059173 \| \| FOXP3 \| 0.162055 \| 0.29079776 \| 1.07763529 \| \| IRGM \| 0.159769 \| 0.29079776 \| 1.02710324 \| \| ITGAE \| 0.162031 \| 0.29079776 \| 0.98535241 \| \| NCR1 \| 0.162084 \| 0.29079776 \| 0.89954789 \| \| SKI \| 0.160269 \| 0.29079776 \| 0.93711201 \| \| CD8B \| 0.164511 \| 0.29428654 \| 0.92099741 \| \| CD19 \| 0.169493 \| 0.29969919 \| 0.74935642 \| \| CR1 \| 0.168561 \| 0.29969919 \| 0.87422952 \| \| HLA.DPA1 \| 0.169502 \| 0.29969919 \| 0.87315519 \| \| IKBKG \| 0.168561 \| 0.29969919 \| 0.94193082 \| \| VTN \| 0.170707 \| 0.30095743 \| 0.92160854 \| \| SCARF1 \| 0.171381 \| 0.30127496 \| 1.45139378 \| \| TOLLIP \| 0.174265 \| 0.30546451 \| 0.94469799 \| \| ITLN1 \| 0.176005 \| 0.30763052 \| 1.1476884 \| \| GNLY \| 0.179619 \| 0.31305026 \| 0.88972414 \| \| EGR2 \| 0.180875 \| 0.31434117 \| 1.44450382 \| \| PDCD1 \| 0.186736 \| 0.323605 \| 0.84438391 \| \| EDNRB \| 0.188065 \| 0.32498484 \| 1.34089848 \| \| S100A8 \| 0.190179 \| 0.32770958 \| 1.18120961 \| \| CSF1 \| 0.193623 \| 0.33270431 \| 0.87894208 \| \| PPIA \| 0.199035 \| 0.34103272 \| 1.25074439 \| \| STAT1 \| 0.199588 \| 0.34103272 \| 0.82554514 \| \| IL4R \| 0.203877 \| 0.34738818 \| 1.23528163 \| \| FKBP5 \| 0.20496 \| 0.34821003 \| 0.88890059 \| \| GFI1 \| 0.205501 \| 0.34821003 \| 0.94422952 \| \| TYK2 \| 0.206592 \| 0.34908898 \| 0.9034715 \| \| KLRC3 \| 0.209884 \| 0.35367193 \| 0.8366476 \| \| LILRB3 \| 0.216018 \| 0.36300545 \| 0.89537441 \| \| FCGR2B \| 0.217147 \| 0.36390019 \| 0.83392641 \| \| CD2 \| 0.219988 \| 0.36765118 \| 0.94258158 \| \| TAP2 \| 0.221706 \| 0.36945722 \| 0.88409787 \| \| TNFRSF1B \| 0.22228 \| 0.36945722 \| 1.19749032 \| \| TP53 \| 0.223432 \| 0.37036283 \| 1.09566746 \| \| BCAP31 \| 0.232212 \| 0.38272745 \| 0.95361954 \| \| C8G \| 0.23338 \| 0.38272745 \| 0.93350594 \| \| IL4 \| 0.232643 \| 0.38272745 \| 0.91201408 \| \| PRKCD \| 0.233401 \| 0.38272745 \| 1.25379718 \| \| LTF \| 0.23699 \| 0.38757078 \| 0.79073599 \| \| CD3EAP \| 0.24763 \| 0.40291069 \| 0.95418499 \| \| LILRA6 \| 0.247691 \| 0.40291069 \| 1.91359298 \| \| CXCR4 \| 0.250498 \| 0.40639303 \| 0.86551876 \| \| B2M \| 0.254273 \| 0.41033474 \| 0.90280042 \| \| TNFRSF11A \| 0.254201 \| 0.41033474 \| 0.74298192 \| \| STAT4 \| 0.264531 \| 0.4257623 \| 0.93657972 \| \| IKZF2 \| 0.271007 \| 0.43389572 \| 1.12282259 \| \| RORC \| 0.27048 \| 0.43389572 \| 0.8406633 \| \| ATG5 \| 0.278415 \| 0.4445894 \| 1.34550337 \| \| DPP4 \| 0.289657 \| 0.4613336 \| 0.97334206 \| \| ATG16L1 \| 0.292796 \| 0.46511865 \| 1.06504958 \| \| CXCR6 \| 0.295033 \| 0.46647775 \| 1.43213056 \| \| IL13 \| 0.295181 \| 0.46647775 \| 0.85225201 \| \| GZMK \| 0.296995 \| 0.46813165 \| 0.93233091 \| \| CD164 \| 0.301243 \| 0.4723862 \| 0.91049185 \| \| NOD2 \| 0.301243 \| 0.4723862 \| 1.29559876 \| \| IL12A \| 0.307525 \| 0.48100064 \| 0.97276741 \| \| FN1 \| 0.310438 \| 0.48431504 \| 0.82616852 \| \| ANKRD22 \| 0.31536 \| 0.48847726 \| 1.38419865 \| \| BAX \| 0.317864 \| 0.48847726 \| 0.94644869 \| \| CCBP2 \| 0.318713 \| 0.48847726 \| 1.0254983 \| \| CD160 \| 0.322005 \| 0.48847726 \| 0.86480094 \| \| CD45RA \| 0.324529 \| 0.48847726 \| 0.98905825 \| \| CD97 \| 0.326022 \| 0.48847726 \| 0.93570684 \| \| CEACAM8 \| 0.320801 \| 0.48847726 \| 0.74039445 \| \| IL19 \| 0.325767 \| 0.48847726 \| 1.00189353 \| \| IL2 \| 0.325767 \| 0.48847726 \| 1.034742 \| \| IL22 \| 0.325767 \| 0.48847726 \| 1.0306703 \| \| IL26 \| 0.320108 \| 0.48847726 \| 0.98735117 \| \| MAPK14 \| 0.327519 \| 0.48847726 \| 0.96051297 \| \| MASP2 \| 0.325767 \| 0.48847726 \| 1.00370646 \| \| PIGR \| 0.320108 \| 0.48847726 \| 0.99101555 \| \| PLA2G2A \| 0.320108 \| 0.48847726 \| 0.98075405 \| \| RAG2 \| 0.320108 \| 0.48847726 \| 0.99101555 \| \| UBE2L3 \| 0.32752 \| 0.48847726 \| 1.00654362 \| \| VCAM1 \| 0.324406 \| 0.48847726 \| 1.02351468 \| \| TLR3 \| 0.329242 \| 0.48865601 \| 1.0528462 \| \| XBP1 \| 0.329022 \| 0.48865601 \| 1.19473529 \| \| TIRAP \| 0.330712 \| 0.48964641 \| 0.84712689 \| \| LILRA5 \| 0.33204 \| 0.49042228 \| 1.14940163 \| \| CASP4 \| 0.335075 \| 0.49225094 \| 1.1406973 \| \| IL1RAP \| 0.335075 \| 0.49225094 \| 1.19323815 \| \| TLR5 \| 0.335699 \| 0.49225094 \| 0.83518517 \| \| HLA.DQA1 \| 0.34947 \| 0.51121511 \| 1.12992421 \| \| LY96 \| 0.360797 \| 0.52517369 \| 0.90837065 \| \| NFATC1 \| 0.361595 \| 0.52517369 \| 0.97400852 \| \| TFRC \| 0.361595 \| 0.52517369 \| 0.94366474 \| \| RELA \| 0.3648 \| 0.52857007 \| 1.20784221 \| \| GUSB \| 0.372888 \| 0.53784839 \| 0.96927289 \| \| XCL1 \| 0.372967 \| 0.53784839 \| 1.01761723 \| \| C2 \| 0.375812 \| 0.54067292 \| 1.2932077 \| \| CIITA \| 0.381735 \| 0.547902 \| 0.88459194 \| \| ITGA2B \| 0.385545 \| 0.55207148 \| 0.8647416 \| \| CFP \| 0.387724 \| 0.55259729 \| 1.11296868 \| \| TAP1 \| 0.387724 \| 0.55259729 \| 0.87414408 \| \| GPI \| 0.389394 \| 0.55368378 \| 1.16969954 \| \| IL18RAP \| 0.391068 \| 0.55477088 \| 1.16180907 \| \| CFB \| 0.396128 \| 0.56007461 \| 1.02194057 \| \| IL28A \| 0.396643 \| 0.56007461 \| 0.9261495 \| \| PDGFB \| 0.398913 \| 0.56197905 \| 1.07397991 \| \| ATG12 \| 0.41275 \| 0.57893067 \| 0.85002784 \| \| CCL13 \| 0.412844 \| 0.57893067 \| 1.02274331 \| \| GATA3 \| 0.417817 \| 0.58411339 \| 0.88218125 \| \| TLR4 \| 0.418455 \| 0.58411339 \| 0.97002504 \| \| CEACAM6 \| 0.420253 \| 0.58528386 \| 0.81710138 \| \| IL2RG \| 0.423713 \| 0.58875838 \| 1.15033494 \| \| CCR6 \| 0.42698 \| 0.59194955 \| 0.98689219 \| \| KLRC1 \| 0.428002 \| 0.59202091 \| 0.99556623 \| \| TAGAP \| 0.436131 \| 0.60190025 \| 0.98777742 \| \| MCL1 \| 0.437922 \| 0.60300772 \| 1.12754186 \| \| CD70 \| 0.442715 \| 0.60686775 \| 0.89480486 \| \| IL11RA \| 0.442156 \| 0.60686775 \| 0.81014778 \| \| CHUK \| 0.446033 \| 0.6080529 \| 0.91815778 \| \| CTSG \| 0.445258 \| 0.6080529 \| 0.88108677 \| \| HRAS \| 0.44657 \| 0.6080529 \| 1.20391874 \| \| SLAMF1 \| 0.468797 \| 0.6368957 \| 1.23558906 \| \| TBP \| 0.472742 \| 0.63940714 \| 1.01935516 \| \| TGFBR1 \| 0.471807 \| 0.63940714 \| 0.92797896 \| \| HLA.DRB1 \| 0.474617 \| 0.64052294 \| 1.12235184 \| \| TNFRSF13B \| 0.479907 \| 0.64623238 \| 1.18562766 \| \| KIR3DL1 \| 0.482274 \| 0.6479893 \| 1.08763539 \| \| KLRF1 \| 0.483841 \| 0.64866596 \| 1.05579116 \| \| ITGAM \| 0.484996 \| 0.64878851 \| 1.15067627 \| \| IFIH1 \| 0.487849 \| 0.651177 \| 1.19095673 \| \| IL15 \| 0.494111 \| 0.65809544 \| 0.99043431 \| \| IRF5 \| 0.500886 \| 0.66473557 \| 1.24508135 \| \| RUNX1 \| 0.501276 \| 0.66473557 \| 1.24443165 \| \| IKZF1 \| 0.504179 \| 0.6671349 \| 1.08122741 \| \| LACTB \| 0.507091 \| 0.66953574 \| 1.12421347 \| \| HLA.DOB \| 0.523646 \| 0.68990078 \| 1.31224854 \| \| CALML4 \| 0.531688 \| 0.69898638 \| 1.02623384 \| \| STAT3 \| 0.536685 \| 0.70403839 \| 1.06416569 \| \| C4BPA \| 0.548442 \| 0.71791764 \| 1.13857395 \| \| BLNK \| 0.551223 \| 0.7189473 \| 1.14149219 \| \| C6 \| 0.553943 \| 0.7189473 \| 1.173026 \| \| NFIL3 \| 0.552816 \| 0.7189473 \| 1.10844562 \| \| ZBTB16 \| 0.553341 \| 0.7189473 \| 0.98897092 \| \| CCL15 \| 0.563694 \| 0.72542899 \| 1.02126035 \| \| CX3CL1 \| 0.563694 \| 0.72542899 \| 1.02102012 \| \| GP1BB \| 0.562819 \| 0.72542899 \| 1.00555879 \| \| IFI35 \| 0.563215 \| 0.72542899 \| 1.28270996 \| \| DEFB103A \| 0.568237 \| 0.72667625 \| 0.99825116 \| \| EOMES \| 0.567464 \| 0.72667625 \| 0.89731847 \| \| LGALS3 \| 0.567123 \| 0.72667625 \| 1.20090831 \| \| HLA.DRB3 \| 0.579526 \| 0.7380185 \| 0.91746943 \| \| SERPING1 \| 0.578631 \| 0.7380185 \| 0.84752451 \| \| BST2 \| 0.584731 \| 0.74155075 \| 0.9955756 \| \| TGFBI \| 0.584731 \| 0.74155075 \| 0.90999782 \| \| SYK \| 0.587865 \| 0.74397853 \| 0.99163273 \| \| TCF4 \| 0.598361 \| 0.75569402 \| 0.95334857 \| \| MAF \| 0.603403 \| 0.76048725 \| 1.10522799 \| \| PTPN2 \| 0.607888 \| 0.76142029 \| 1.14269312 \| \| PTPN22 \| 0.607881 \| 0.76142029 \| 1.11340386 \| \| TRAF6 \| 0.607888 \| 0.76142029 \| 0.97447002 \| \| LITAF \| 0.612142 \| 0.7651775 \| 0.9596249 \| \| ATG10 \| 0.622806 \| 0.77375084 \| 1.18880163 \| \| CD7 \| 0.621761 \| 0.77375084 \| 1.19495062 \| \| CR2 \| 0.622305 \| 0.77375084 \| 1.07204763 \| \| LTA \| 0.628935 \| 0.77977713 \| 1.13401051 \| \| LILRA4 \| 0.635623 \| 0.78647065 \| 0.89674518 \| \| S100A9 \| 0.643367 \| 0.79444103 \| 1.08515866 \| \| HLA.DQB1 \| 0.646359 \| 0.79652321 \| 1.12634751 \| \| ILF3 \| 0.647726 \| 0.79659851 \| 1.02292048 \| \| CFH \| 0.657738 \| 0.80404846 \| 0.85253738 \| \| GPR183 \| 0.657576 \| 0.80404846 \| 1.12008716 \| \| IRF7 \| 0.656479 \| 0.80404846 \| 1.01799862 \| \| C5 \| 0.661618 \| 0.80717396 \| 0.91560548 \| \| C1QA \| 0.664184 \| 0.80868711 \| 1.02361437 \| \| ICAM4 \| 0.675353 \| 0.82064807 \| 0.92435504 \| \| CD86 \| 0.678564 \| 0.82291062 \| 1.18018743 \| \| FADD \| 0.68175 \| 0.82503285 \| 0.90721852 \| \| MAP4K4 \| 0.683019 \| 0.82503285 \| 1.1516657 \| \| CASP3 \| 0.68748 \| 0.82714556 \| 1.00903177 \| \| CTSC \| 0.686365 \| 0.82714556 \| 1.05312844 \| \| BST1 \| 0.691955 \| 0.83089085 \| 1.02081257 \| \| IFI16 \| 0.698686 \| 0.83568325 \| 0.98304134 \| \| PTPN6 \| 0.698686 \| 0.83568325 \| 0.9633485 \| \| NOTCH2 \| 0.700935 \| 0.83673258 \| 1.01019156 \| \| C1QBP \| 0.703186 \| 0.8377802 \| 1.0741087 \| \| FCGR1A.B \| 0.708183 \| 0.84208895 \| 1.17109816 \| \| ACTA2 \| 0.715336 \| 0.8479308 \| 1.01648195 \| \| PPARG \| 0.715876 \| 0.8479308 \| 0.92670496 \| \| CD59 \| 0.725842 \| 0.85806903 \| 1.04673214 \| \| KCNJ15 \| 0.733824 \| 0.86582716 \| 1.23034166 \| \| ARG1 \| 0.764021 \| 0.89971585 \| 0.86905718 \| \| FCGR2A \| 0.771861 \| 0.90371441 \| 0.97498383 \| \| IKBKB \| 0.76948 \| 0.90371441 \| 1.07116186 \| \| PTPRC_all \| 0.771861 \| 0.90371441 \| 0.96544241 \| \| IL1RL2 \| 0.776761 \| 0.90592567 \| 1.02843534 \| \| KIR3DL2 \| 0.778205 \| 0.90592567 \| 1.02909338 \| \| MRC1 \| 0.777206 \| 0.90592567 \| 1.17974986 \| \| APP \| 0.787 \| 0.90713791 \| 1.03746308 \| \| IRF4 \| 0.7835 \| 0.90713791 \| 1.03826556 \| \| LCP2 \| 0.785833 \| 0.90713791 \| 1.09603206 \| \| LILRB5 \| 0.781927 \| 0.90713791 \| 0.99227114 \| \| MX1 \| 0.788169 \| 0.90713791 \| 1.09320427 \| \| PRDM1 \| 0.788169 \| 0.90713791 \| 1.04931054 \| \| PLA2G2E \| 0.800459 \| 0.919548 \| 1.00293577 \| \| B3GAT1 \| 0.807183 \| 0.9203395 \| 0.98248658 \| \| CUL9 \| 0.806889 \| 0.9203395 \| 1.19257914 \| \| IKZF3 \| 0.80457 \| 0.9203395 \| 0.91413174 \| \| LOC389386 \| 0.80457 \| 0.9203395 \| 0.97716494 \| \| BATF2 \| 0.809272 \| 0.92099985 \| 1.08594497 \| \| STAT5A \| 0.817517 \| 0.9286506 \| 1.05717939 \| \| GBP6 \| 0.821601 \| 0.93155504 \| 0.89799605 \| \| TBK1 \| 0.823419 \| 0.93188421 \| 1.12946847 \| \| CD8A \| 0.828149 \| 0.93204961 \| 0.968047 \| \| CTLA4_all \| 0.825783 \| 0.93204961 \| 1.11452424 \| \| TNFSF4 \| 0.826835 \| 0.93204961 \| 1.12303898 \| \| PSMD7 \| 0.832884 \| 0.93393243 \| 0.98874898 \| \| TAPBP \| 0.832884 \| 0.93393243 \| 0.98977836 \| \| PDGFRB \| 0.83849 \| 0.9384593 \| 0.97786716 \| \| PTGER4 \| 0.839998 \| 0.9384593 \| 1.06076472 \| \| PSMB7 \| 0.856647 \| 0.95356692 \| 1.00243227 \| \| TNFRSF14 \| 0.855455 \| 0.95356692 \| 1.02925253 \| \| ITGAX \| 0.868579 \| 0.96508778 \| 1.04980553 \| \| CD45R0 \| 0.87336 \| 0.96512609 \| 1.02663383 \| \| FCGR2A.C \| 0.870969 \| 0.96512609 \| 1.04707472 \| \| ZEB1 \| 0.872163 \| 0.96512609 \| 1.10315843 \| \| IL18 \| 0.890158 \| 0.98191027 \| 1.01696117 \| \| GZMB \| 0.897333 \| 0.98803814 \| 0.98751913 \| \| ITGB1 \| 0.899735 \| 0.98889793 \| 1.02570048 \| \| TAL1 \| 0.902935 \| 0.99063013 \| 0.96736839 \| \| CCL24 \| 0.906946 \| 0.99146427 \| 1.16864602 \| \| TNFRSF17 \| 0.90635 \| 0.99146427 \| 1.01929115 \| \| EGR1 \| 0.911509 \| 0.99447646 \| 0.97408191 \| \| KLRD1 \| 0.912962 \| 0.99447646 \| 1.13724572 \| \| BCL10 \| 0.981834 \| 1 \| 0.99146668 \| \| C1R \| 1 \| 1 \| 1 \| \| C1S \| 1 \| 1 \| 1 \| \| C7 \| 1 \| 1 \| 1 \| \| C8A \| 1 \| 1 \| 1 \| \| C8B \| 1 \| 1 \| 1 \| \| C9 \| 1 \| 1 \| 1 \| \| CCL11 \| 1 \| 1 \| 1 \| \| CCL16 \| 1 \| 1 \| 1 \| \| CCL26 \| 1 \| 1 \| 1 \| \| CCR8 \| 1 \| 1 \| 1 \| \| CCRL1 \| 0.993459 \| 1 \| 0.9769923 \| \| CD1A \| 1 \| 1 \| 1 \| \| CD34 \| 1 \| 1 \| 1 \| \| CD48 \| 0.946747 \| 1 \| 1.05431641 \| \| CDH5 \| 1 \| 1 \| 1 \| \| CFI \| 1 \| 1 \| 1 \| \| CLU \| 1 \| 1 \| 1 \| \| CXCL12 \| 0.976741 \| 1 \| 0.97463052 \| \| DEFB103B \| 1 \| 1 \| 1 \| \| GBP1 \| 0.950373 \| 1 \| 1.02038819 \| \| HAMP \| 1 \| 1 \| 1 \| \| HFE \| 1 \| 1 \| 1 \| \| IFNA2 \| 1 \| 1 \| 1 \| \| IFNB1 \| 1 \| 1 \| 1 \| \| IFNGR1 \| 0.929841 \| 1 \| 1.01551595 \| \| IL17A \| 1 \| 1 \| 1 \| \| IL17B \| 1 \| 1 \| 1 \| \| IL18R1 \| 0.956418 \| 1 \| 0.99622314 \| \| IL21 \| 1 \| 1 \| 1 \| \| IL22RA2 \| 1 \| 1 \| 1 \| \| IL27 \| 0.996179 \| 1 \| 1.05060234 \| \| IL29 \| 1 \| 1 \| 1 \| \| IL2RB \| 0.952791 \| 1 \| 1.01714186 \| \| IL3 \| 0.993459 \| 1 \| 0.98962627 \| \| IL5 \| 1 \| 1 \| 1 \| \| IL9 \| 1 \| 1 \| 1 \| \| IRAK4 \| 0.956418 \| 1 \| 1.07324764 \| \| ITGA5 \| 0.943122 \| 1 \| 1.01115958 \| \| ITLN2 \| 1 \| 1 \| 1 \| \| KIR3DL3 \| 1 \| 1 \| 1 \| \| KLRAP1 \| 0.951339 \| 1 \| 0.9944749 \| \| MAPKAPK2 \| 0.969727 \| 1 \| 1.06804727 \| \| MASP1 \| 1 \| 1 \| 1 \| \| MBL2 \| 1 \| 1 \| 1 \| \| MUC1 \| 1 \| 1 \| 1.03402667 \| \| MYD88 \| 0.996366 \| 1 \| 0.99793393 \| \| NOS2 \| 0.993459 \| 1 \| 0.97639668 \| \| TLR7 \| 0.979226 \| 1 \| 1.01148386 \| \| TNFSF11 \| 1 \| 1 \| 1.01017007 \| |
| --- | --- | --- | --- | --- | --- | --- | --- | --- | --- | --- | --- | --- | --- | --- | --- | --- | --- | --- | --- | --- | --- | --- | --- | --- | --- | --- | --- | --- | --- | --- | --- | --- | --- | --- | --- | --- | --- | --- | --- | --- | --- | --- | --- | --- | --- | --- | --- | --- | --- | --- | --- | --- | --- | --- | --- | --- | --- | --- | --- | --- | --- | --- | --- | --- | --- | --- | --- | --- | --- | --- | --- | --- | --- | --- | --- | --- | --- | --- | --- | --- | --- | --- | --- | --- | --- | --- | --- | --- | --- | --- | --- | --- | --- | --- | --- | --- | --- | --- | --- | --- | --- | --- | --- | --- | --- | --- | --- | --- | --- | --- | --- | --- | --- | --- | --- | --- | --- | --- | --- | --- | --- | --- | --- | --- | --- | --- | --- | --- | --- | --- | --- | --- | --- | --- | --- | --- | --- | --- | --- | --- | --- | --- | --- | --- | --- | --- | --- | --- | --- | --- | --- | --- | --- | --- | --- | --- | --- | --- | --- | --- | --- | --- | --- | --- | --- | --- | --- | --- | --- | --- | --- | --- | --- | --- | --- | --- | --- | --- | --- | --- | --- | --- | --- | --- | --- | --- | --- | --- | --- | --- | --- | --- | --- | --- | --- | --- | --- | --- | --- | --- | --- | --- | --- | --- | --- | --- | --- | --- | --- | --- | --- | --- | --- | --- | --- | --- | --- | --- | --- | --- | --- | --- | --- | --- | --- | --- | --- | --- | --- | --- | --- | --- | --- | --- | --- | --- | --- | --- | --- | --- | --- | --- | --- | --- | --- | --- | --- | --- | --- | --- | --- | --- | --- | --- | --- | --- | --- | --- | --- | --- | --- | --- | --- | --- | --- | --- | --- | --- | --- | --- | --- | --- | --- | --- | --- | --- | --- | --- | --- | --- | --- | --- | --- | --- | --- | --- | --- | --- | --- | --- | --- | --- | --- | --- | --- | --- | --- | --- | --- | --- | --- | --- | --- | --- | --- | --- | --- | --- | --- | --- | --- | --- | --- | --- | --- | --- | --- | --- | --- | --- | --- | --- | --- | --- | --- | --- | --- | --- | --- | --- | --- | --- | --- | --- | --- | --- | --- | --- | --- | --- | --- | --- | --- | --- | --- | --- | --- | --- | --- | --- | --- | --- | --- | --- | --- | --- | --- | --- | --- | --- | --- | --- | --- | --- | --- | --- | --- | --- | --- | --- | --- | --- | --- | --- | --- | --- | --- | --- | --- | --- | --- | --- | --- | --- | --- | --- | --- | --- | --- | --- | --- | --- | --- | --- | --- | --- | --- | --- | --- | --- | --- | --- | --- | --- | --- | --- | --- | --- | --- | --- | --- | --- | --- | --- | --- | --- | --- | --- | --- | --- | --- | --- | --- | --- | --- | --- | --- | --- | --- | --- | --- | --- | --- | --- | --- | --- | --- | --- | --- | --- | --- | --- | --- | --- | --- | --- | --- | --- | --- | --- | --- | --- | --- | --- | --- | --- | --- | --- | --- | --- | --- | --- | --- | --- | --- | --- | --- | --- | --- | --- | --- | --- | --- | --- | --- | --- | --- | --- | --- | --- | --- | --- | --- | --- | --- | --- | --- | --- | --- | --- | --- | --- | --- | --- | --- | --- | --- | --- | --- | --- | --- | --- | --- | --- | --- | --- | --- | --- | --- | --- | --- | --- | --- | --- | --- | --- | --- | --- | --- | --- | --- | --- | --- | --- | --- | --- | --- | --- | --- | --- | --- | --- | --- | --- | --- | --- | --- | --- | --- | --- | --- | --- | --- | --- | --- | --- | --- | --- | --- | --- | --- | --- | --- | --- | --- | --- | --- | --- | --- | --- | --- | --- | --- | --- | --- | --- | --- | --- | --- | --- | --- | --- | --- | --- | --- | --- | --- | --- | --- | --- | --- | --- | --- | --- | --- | --- | --- | --- | --- | --- | --- | --- | --- | --- | --- | --- | --- | --- | --- | --- | --- | --- | --- | --- | --- | --- | --- | --- | --- | --- | --- | --- | --- | --- | --- | --- | --- | --- | --- | --- | --- | --- | --- | --- | --- | --- | --- | --- | --- | --- | --- | --- | --- | --- | --- | --- | --- | --- | --- | --- | --- | --- | --- | --- | --- | --- | --- | --- | --- | --- | --- | --- | --- | --- | --- | --- | --- | --- | --- | --- | --- | --- | --- | --- | --- | --- | --- | --- | --- | --- | --- | --- | --- | --- | --- | --- | --- | --- | --- | --- | --- | --- | --- | --- | --- | --- | --- | --- | --- | --- | --- | --- | --- | --- | --- | --- | --- | --- | --- | --- | --- | --- | --- | --- | --- | --- | --- | --- | --- | --- | --- | --- | --- | --- | --- | --- | --- | --- | --- | --- | --- | --- | --- | --- | --- | --- | --- | --- | --- | --- | --- | --- | --- | --- | --- | --- | --- | --- | --- | --- | --- | --- | --- | --- | --- | --- | --- | --- | --- | --- | --- | --- | --- | --- | --- | --- | --- | --- | --- | --- | --- | --- | --- | --- | --- | --- | --- | --- | --- | --- | --- | --- | --- | --- | --- | --- | --- | --- | --- | --- | --- | --- | --- | --- | --- | --- | --- | --- | --- | --- | --- | --- | --- | --- | --- | --- | --- | --- | --- | --- | --- | --- | --- | --- | --- | --- | --- | --- | --- | --- | --- | --- | --- | --- | --- | --- | --- | --- | --- | --- | --- | --- | --- | --- | --- | --- | --- | --- | --- | --- | --- | --- | --- | --- | --- | --- | --- | --- | --- | --- | --- | --- | --- | --- | --- | --- | --- | --- | --- | --- | --- | --- | --- | --- | --- | --- | --- | --- | --- | --- | --- | --- | --- | --- | --- | --- | --- | --- | --- | --- | --- | --- | --- | --- | --- | --- | --- | --- | --- | --- | --- | --- | --- | --- | --- | --- | --- | --- | --- | --- | --- | --- | --- | --- | --- | --- | --- | --- | --- | --- | --- | --- | --- | --- | --- | --- | --- | --- | --- | --- | --- | --- | --- | --- | --- | --- | --- | --- | --- | --- | --- | --- | --- | --- | --- | --- | --- | --- | --- | --- | --- | --- | --- | --- | --- | --- | --- | --- | --- | --- | --- | --- | --- | --- | --- | --- | --- | --- | --- | --- | --- | --- | --- | --- | --- | --- | --- | --- | --- | --- | --- | --- | --- | --- | --- | --- | --- | --- | --- | --- | --- | --- | --- | --- | --- | --- | --- | --- | --- | --- | --- | --- | --- | --- | --- | --- | --- | --- | --- | --- | --- | --- | --- | --- | --- | --- | --- | --- | --- | --- | --- | --- | --- | --- | --- | --- | --- | --- | --- | --- | --- | --- | --- | --- | --- | --- | --- | --- | --- | --- | --- | --- | --- | --- | --- | --- | --- | --- | --- | --- | --- | --- | --- | --- | --- | --- | --- | --- | --- | --- | --- | --- | --- | --- | --- | --- | --- | --- | --- | --- | --- | --- | --- | --- | --- | --- | --- | --- | --- | --- | --- | --- | --- | --- | --- | --- | --- | --- | --- | --- | --- | --- | --- | --- | --- | --- | --- | --- | --- | --- | --- | --- | --- | --- | --- | --- | --- | --- | --- | --- | --- | --- | --- | --- | --- | --- | --- | --- | --- | --- | --- | --- | --- | --- | --- | --- | --- | --- | --- | --- | --- | --- | --- | --- | --- | --- | --- | --- | --- | --- | --- | --- | --- | --- | --- | --- | --- | --- | --- | --- | --- | --- | --- | --- | --- | --- | --- | --- | --- | --- | --- | --- | --- | --- | --- | --- | --- | --- | --- | --- | --- | --- | --- | --- | --- | --- | --- | --- | --- | --- | --- | --- | --- | --- | --- | --- | --- | --- | --- | --- | --- | --- | --- | --- | --- | --- | --- | --- | --- | --- | --- | --- | --- | --- | --- | --- | --- | --- | --- | --- | --- | --- | --- | --- | --- | --- | --- | --- | --- | --- | --- | --- | --- | --- | --- | --- | --- | --- | --- | --- | --- | --- | --- | --- | --- | --- | --- | --- | --- | --- | --- | --- | --- | --- | --- | --- | --- | --- | --- | --- | --- | --- | --- | --- | --- | --- | --- | --- | --- | --- | --- | --- | --- | --- | --- | --- | --- | --- | --- | --- | --- | --- | --- | --- | --- | --- | --- | --- | --- | --- | --- | --- | --- | --- | --- | --- | --- | --- | --- | --- | --- | --- | --- | --- | --- | --- | --- | --- | --- | --- | --- | --- | --- | --- | --- | --- | --- | --- | --- | --- | --- | --- | --- | --- | --- | --- | --- | --- | --- | --- | --- | --- | --- | --- | --- | --- | --- | --- | --- | --- | --- | --- | --- | --- | --- | --- | --- | --- | --- | --- | --- | --- | --- | --- | --- | --- | --- | --- | --- | --- | --- | --- | --- | --- | --- | --- | --- | --- | --- | --- | --- | --- | --- | --- | --- | --- | --- | --- | --- | --- | --- | --- | --- | --- | --- | --- | --- | --- | --- | --- | --- | --- | --- | --- | --- | --- | --- | --- | --- | --- | --- | --- | --- | --- | --- | --- | --- | --- | --- | --- | --- | --- | --- | --- | --- | --- | --- | --- | --- | --- | --- | --- | --- | --- | --- | --- | --- | --- | --- | --- | --- | --- | --- | --- | --- | --- | --- | --- | --- | --- | --- | --- | --- | --- | --- | --- | --- | --- | --- | --- | --- | --- | --- | --- | --- | --- | --- | --- | --- | --- | --- | --- | --- | --- | --- | --- | --- | --- | --- | --- | --- | --- | --- | --- | --- | --- | --- | --- | --- | --- | --- | --- | --- | --- | --- | --- | --- | --- | --- | --- | --- | --- | --- | --- | --- | --- | --- | --- | --- | --- | --- | --- | --- | --- | --- | --- | --- | --- | --- | --- | --- | --- | --- | --- | --- | --- | --- | --- | --- | --- | --- | --- | --- | --- | --- | --- | --- | --- | --- | --- | --- | --- | --- | --- | --- | --- | --- | --- | --- | --- | --- | --- | --- | --- | --- | --- | --- | --- | --- | --- | --- | --- | --- | --- | --- | --- | --- | --- | --- | --- | --- | --- | --- | --- | --- | --- | --- | --- | --- | --- | --- | --- | --- | --- | --- | --- | --- | --- | --- | --- | --- | --- | --- | --- | --- | --- | --- | --- | --- | --- | --- | --- | --- | --- | --- | --- | --- | --- | --- | --- | --- | --- | --- | --- | --- | --- | --- | --- | --- | --- | --- | --- | --- | --- | --- | --- | --- | --- | --- | --- | --- | --- | --- | --- | --- | --- | --- | --- | --- | --- | --- | --- | --- | --- | --- | --- | --- | --- | --- | --- | --- | --- | --- | --- | --- | --- | --- | --- | --- | --- | --- | --- | --- | --- | --- | --- | --- | --- | --- | --- | --- | --- | --- | --- | --- | --- | --- | --- | --- | --- | --- | --- | --- | --- | --- | --- | --- | --- | --- | --- | --- | --- | --- | --- | --- | --- | --- | --- | --- | --- | --- | --- | --- | --- | --- | --- | --- | --- | --- | --- | --- | --- | --- | --- | --- | --- | --- | --- | --- | --- | --- | --- | --- | --- | --- | --- | --- | --- | --- | --- | --- | --- | --- | --- | --- | --- | --- | --- | --- | --- | --- | --- | --- | --- | --- | --- | --- | --- | --- | --- | --- | --- | --- | --- | --- | --- | --- | --- | --- | --- | --- | --- | --- | --- | --- | --- | --- | --- | --- | --- | --- | --- | --- | --- | --- | --- | --- | --- | --- | --- | --- | --- | --- | --- | --- | --- | --- | --- | --- | --- | --- | --- | --- | --- | --- | --- | --- | --- | --- | --- | --- | --- | --- | --- | --- | --- | --- | --- | --- | --- | --- | --- | --- | --- | --- | --- | --- | --- | --- | --- | --- | --- | --- | --- | --- | --- | --- | --- | --- | --- | --- | --- | --- | --- | --- | --- | --- | --- | --- | --- | --- | --- | --- | --- | --- | --- | --- | --- | --- | --- | --- | --- | --- | --- | --- | --- | --- | --- | --- | --- | --- | --- | --- | --- | --- | --- | --- | --- | --- | --- | --- | --- | --- | --- | --- | --- | --- | --- | --- | --- | --- | --- | --- | --- | --- | --- | --- | --- | --- | --- | --- | --- | --- | --- | --- | --- | --- | --- | --- | --- | --- | --- | --- | --- | --- | --- | --- | --- | --- | --- | --- | --- | --- | --- | --- | --- | --- | --- | --- | --- | --- | --- | --- | --- | --- | --- | --- | --- | --- | --- | --- | --- | --- | --- | --- | --- | --- | --- | --- | --- | --- | --- | --- | --- | --- | --- | --- | --- | --- | --- | --- | --- | --- | --- | --- | --- | --- | --- | --- | --- | --- | --- | --- | --- | --- | --- | --- | --- | --- | --- | --- | --- | --- | --- | --- | --- | --- | --- | --- | --- | --- | --- | --- | --- | --- | --- | --- | --- | --- | --- | --- | --- | --- | --- | --- | --- | --- | --- | --- | --- | --- | --- | --- | --- | --- | --- | --- | --- | --- | --- | --- | --- | --- | --- | --- | --- | --- | --- | --- | --- | --- | --- | --- | --- | --- | --- | --- | --- | --- | --- | --- | --- | --- | --- | --- | --- | --- | --- | --- | --- | --- | --- | --- | --- | --- | --- | --- | --- | --- | --- | --- | --- | --- | --- | --- | --- | --- | --- | --- | --- | --- | --- | --- | --- | --- | --- | --- | --- | --- | --- | --- | --- | --- | --- | --- | --- | --- | --- | --- | --- | --- | --- | --- | --- | --- | --- | --- | --- | --- | --- | --- | --- | --- | --- | --- | --- | --- | --- | --- | --- | --- | --- | --- | --- | --- | --- | --- | --- | --- | --- | --- | --- | --- | --- | --- | --- | --- | --- | --- | --- | --- | --- | --- | --- | --- | --- | --- | --- | --- | --- | --- | --- | --- | --- | --- | --- | --- | --- | --- | --- | --- | --- | --- | --- | --- | --- | --- | --- | --- | --- | --- | --- | --- | --- | --- | --- | --- | --- | --- | --- | --- | --- | --- | --- | --- | --- | --- | --- | --- | --- | --- | --- | --- | --- | --- | --- | --- | --- | --- | --- | --- | --- | --- | --- | --- | --- | --- | --- | --- | --- | --- | --- | --- | --- | --- | --- | --- | --- | --- | --- | --- | --- | --- | --- | --- | --- | --- | --- | --- | --- | --- | --- | --- | --- | --- | --- | --- | --- | --- | --- | --- | --- | --- | --- | --- | --- | --- | --- | --- | --- | --- | --- | --- | --- | --- | --- | --- | --- | --- | --- | --- | --- | --- | --- | --- | --- | --- | --- | --- | --- | --- | --- | --- | --- | --- | --- | --- | --- | --- | --- | --- | --- | --- | --- | --- | --- | --- | --- | --- | --- | --- | --- | --- | --- | --- | --- | --- | --- | --- | --- | --- | --- | --- | --- | --- | --- | --- | --- | --- | --- | --- | --- | --- | --- | --- | --- | --- | --- | --- | --- | --- | --- | --- | --- | --- | --- | --- | --- | --- | --- | --- | --- | --- | --- | --- | --- | --- | --- | --- | --- | --- | --- | --- | --- | --- | --- | --- | --- | --- | --- | --- | --- | --- | --- | --- | --- | --- | --- | --- | --- | --- | --- | --- | --- | --- | --- | --- | --- | --- | --- | --- | --- | --- | --- | --- | --- | --- | --- | --- | --- | --- | --- | --- | --- | --- | --- | --- | --- | --- | --- | --- | --- | --- | --- | --- | --- | --- | --- | --- | --- | --- | --- | --- | --- | --- | --- | --- | --- | --- | --- | --- | --- | --- | --- | --- | --- | --- | --- | --- | --- | --- | --- | --- | --- | --- | --- | --- | --- | --- | --- | --- | --- | --- | --- | --- | --- | --- | --- | --- | --- | --- | --- | --- | --- | --- | --- | --- | --- | --- | --- | --- | --- | --- | --- | --- | --- | --- | --- | --- | --- | --- | --- | --- | --- | --- | --- | --- | --- | --- | --- | --- | --- | --- | --- | --- | --- | --- | --- | --- | --- | --- | --- | --- | --- | --- | --- | --- | --- | --- | --- | --- | --- | --- | --- | --- | --- | --- | --- | --- | --- | --- | --- | --- | --- |

**Supplementary Table S7**. Comparison between Null and IL-1b-induced genes, showing p-value, q-value and fold change.

| \|  \| **Pre-Tx (V1)** \| \| \| **Post-Tx (V2)** \| \| \| \| --- \| --- \| --- \| --- \| --- \| --- \| --- \| \| **Gene** \| **p-value** \| **q-value** \| **Fold change** \| **p-value** \| **q-value** \| **Fold change** \| \| RELB \| 8.61E-12 \| 9.21E-10 \| 4.89 \| 0.0768 \| 0.29 \| 1.34 \| \| ICAM1 \| 1.11E-10 \| 1.98E-09 \| 6.52 \| 0.0719 \| 0.29 \| 1.56 \| \| IRAK3 \| 1.11E-10 \| 1.98E-09 \| 6.58 \| 0.0282 \| 0.29 \| 1.68 \| \| NFKB2 \| 8.59E-11 \| 1.98E-09 \| 4.06 \| 0.0548 \| 0.29 \| 1.52 \| \| NFKBIA \| 1.11E-10 \| 1.98E-09 \| 5.83 \| 0.0383 \| 0.29 \| 1.57 \| \| TNFAIP3 \| 3.84E-11 \| 1.98E-09 \| 5.52 \| 0.0241 \| 0.29 \| 1.58 \| \| NFKB1 \| 1.82E-10 \| 2.79E-09 \| 3.84 \| 0.0819 \| 0.29 \| 1.53 \| \| ATG7 \| 2.32E-10 \| 3.11E-09 \| 4.74 \| 0.0719 \| 0.29 \| 1.47 \| \| CLEC5A \| 4.66E-10 \| 5.54E-09 \| 8.31 \| 0.0930 \| 0.29 \| 1.72 \| \| CDKN1A \| 5.83E-10 \| 6.24E-09 \| 8.82 \| 0.0629 \| 0.29 \| 1.88 \| \| BCL3 \| 1.12E-09 \| 9.21E-09 \| 5.82 \| 0.1957 \| 0.45 \| 1.26 \| \| CCRL2 \| 1.12E-09 \| 9.21E-09 \| 10.45 \| 0.0305 \| 0.29 \| 2.41 \| \| IL8 \| 1.12E-09 \| 9.21E-09 \| 9.53 \| 0.0261 \| 0.29 \| 2.24 \| \| NFKBIZ \| 1.38E-09 \| 1.06E-08 \| 4.79 \| 0.0548 \| 0.29 \| 1.53 \| \| SRC \| 2.55E-09 \| 1.82E-08 \| 4.62 \| 0.0873 \| 0.29 \| 1.61 \| \| TICAM1 \| 4.58E-09 \| 3.06E-08 \| 4.21 \| 0.2060 \| 0.45 \| 1.42 \| \| BATF \| 5.54E-09 \| 3.29E-08 \| 2.50 \| 0.6751 \| 0.87 \| 0.99 \| \| IL1RN \| 5.54E-09 \| 3.29E-08 \| 9.93 \| 0.0873 \| 0.29 \| 2.40 \| \| IRAK2 \| 6.68E-09 \| 3.57E-08 \| 9.63 \| 0.0412 \| 0.29 \| 2.30 \| \| JAK3 \| 6.68E-09 \| 3.57E-08 \| 2.42 \| 1.0000 \| 1.00 \| 1.02 \| \| CTSS \| 9.65E-09 \| 4.49E-08 \| 3.33 \| 0.5340 \| 0.78 \| 1.17 \| \| PTGS2 \| 9.65E-09 \| 4.49E-08 \| 18.16 \| 0.0768 \| 0.29 \| 3.38 \| \| TNFAIP6 \| 9.65E-09 \| 4.49E-08 \| 5.42 \| 0.0511 \| 0.29 \| 1.83 \| \| CXCL2 \| 1.38E-08 \| 6.17E-08 \| 12.59 \| 0.0412 \| 0.29 \| 2.29 \| \| MARCO \| 2.54E-08 \| 1.09E-07 \| 20.91 \| 0.4004 \| 0.70 \| 1.46 \| \| BID \| 2.77E-08 \| 1.14E-07 \| 2.99 \| 0.2591 \| 0.52 \| 1.75 \| \| CCL20 \| 3.27E-08 \| 1.30E-07 \| 13.80 \| 0.0930 \| 0.29 \| 2.11 \| \| PLAU \| 4.56E-08 \| 1.68E-07 \| 24.71 \| 0.0099 \| 0.29 \| 6.23 \| \| SOCS3 \| 4.56E-08 \| 1.68E-07 \| 3.98 \| 0.2765 \| 0.55 \| 1.36 \| \| TRAF1 \| 5.37E-08 \| 1.91E-07 \| 3.27 \| 0.0873 \| 0.29 \| 1.41 \| \| CXCL1 \| 7.40E-08 \| 2.55E-07 \| 5.37 \| 0.0930 \| 0.29 \| 1.70 \| \| CD14 \| 1.86E-07 \| 6.23E-07 \| 3.02 \| 0.2514 \| 0.52 \| 1.17 \| \| TNF \| 2.89E-07 \| 9.39E-07 \| 3.22 \| 0.0673 \| 0.29 \| 1.57 \| \| CD46 \| 5.11E-07 \| 1.61E-06 \| 2.79 \| 0.9424 \| 0.98 \| 1.04 \| \| CASP8 \| 5.88E-07 \| 1.80E-06 \| 2.23 \| 0.9654 \| 0.98 \| 1.01 \| \| CD83 \| 6.74E-07 \| 1.95E-06 \| 4.23 \| 0.0819 \| 0.29 \| 1.70 \| \| POU2F2 \| 6.74E-07 \| 1.95E-06 \| 2.46 \| 0.3314 \| 0.62 \| 1.26 \| \| KCNJ2 \| 8.85E-07 \| 2.43E-06 \| 3.93 \| 0.1333 \| 0.35 \| 1.74 \| \| TNFSF15 \| 8.85E-07 \| 2.43E-06 \| 12.98 \| 0.0511 \| 0.29 \| 2.63 \| \| CSF3R \| 2.50E-06 \| 6.69E-06 \| 3.60 \| 0.6751 \| 0.87 \| 0.92 \| \| IL1B \| 3.63E-06 \| 9.46E-06 \| 5.83 \| 0.1333 \| 0.35 \| 1.81 \| \| CCL3 \| 4.62E-06 \| 1.18E-05 \| 6.01 \| 0.0511 \| 0.29 \| 2.26 \| \| FCER1A \| 4.93E-06 \| 1.23E-05 \| 0.09 \| 0.5102 \| 0.78 \| 1.80 \| \| SLAMF7 \| 5.21E-06 \| 1.27E-05 \| 3.80 \| 0.1258 \| 0.35 \| 1.81 \| \| LILRB4 \| 5.87E-06 \| 1.39E-05 \| 2.64 \| 0.3462 \| 0.63 \| 1.30 \| \| CD274 \| 6.60E-06 \| 1.50E-05 \| 5.95 \| 0.0816 \| 0.29 \| 4.00 \| \| FER1L3 \| 6.60E-06 \| 1.50E-05 \| 4.14 \| 0.8060 \| 0.95 \| 0.75 \| \| S1PR1 \| 9.34E-06 \| 2.08E-05 \| 0.34 \| 0.7837 \| 0.93 \| 1.19 \| \| CD36 \| 1.17E-05 \| 2.51E-05 \| 2.98 \| 0.2514 \| 0.52 \| 1.34 \| \| CXCR2 \| 1.17E-05 \| 2.51E-05 \| 3.61 \| 0.6751 \| 0.87 \| 0.92 \| \| CD244 \| 2.81E-05 \| 5.89E-05 \| 1.93 \| 0.9424 \| 0.98 \| 1.10 \| \| ICAM5 \| 3.93E-05 \| 8.09E-05 \| 12.63 \| 0.0149 \| 0.29 \| 3.74 \| \| C3 \| 4.71E-05 \| 9.52E-05 \| 4.26 \| 0.4648 \| 0.75 \| 1.35 \| \| IL16 \| 7.03E-05 \| 0.0001 \| 1.93 \| 0.8738 \| 0.97 \| 0.98 \| \| CCL7 \| 0.0001 \| 0.0002 \| 2.68 \| 0.6268 \| 0.86 \| 1.47 \| \| CD40 \| 0.0002 \| 0.0003 \| 1.75 \| 0.1186 \| 0.33 \| 1.26 \| \| CCND3 \| 0.0002 \| 0.0004 \| 2.16 \| 0.8151 \| 0.95 \| 0.94 \| \| NFATC3 \| 0.0002 \| 0.0004 \| 1.67 \| 0.7837 \| 0.93 \| 1.02 \| \| CYBB \| 0.0002 \| 0.0004 \| 1.58 \| 0.9195 \| 0.98 \| 1.02 \| \| IKBKE \| 0.0002 \| 0.0004 \| 1.56 \| 0.5532 \| 0.80 \| 1.13 \| \| IL6 \| 0.0003 \| 0.0006 \| 4.94 \| 0.0378 \| 0.29 \| 3.96 \| \| CCL4 \| 0.0004 \| 0.0008 \| 3.22 \| 0.0383 \| 0.29 \| 2.15 \| \| IFIT2 \| 0.0007 \| 0.0011 \| 3.28 \| 0.9650 \| 0.98 \| 1.32 \| \| CASP2 \| 0.0010 \| 0.0017 \| 1.59 \| 0.8966 \| 0.97 \| 1.01 \| \| IL7R \| 0.0013 \| 0.0021 \| 1.46 \| 0.5340 \| 0.78 \| 1.13 \| \| IL1A \| 0.0016 \| 0.0026 \| 3.41 \| 0.1186 \| 0.33 \| 2.50 \| \| LILRB1 \| 0.0023 \| 0.0036 \| 1.69 \| 0.1118 \| 0.33 \| 1.70 \| \| CCL19 \| 0.0028 \| 0.0044 \| 0.24 \| 0.2195 \| 0.47 \| 1.62 \| \| LAIR1 \| 0.0032 \| 0.0050 \| 1.51 \| 0.8966 \| 0.97 \| 0.95 \| \| SELE \| 0.0035 \| 0.0053 \| 2.58 \| 0.1408 \| 0.35 \| 1.49 \| \| SELL \| 0.0046 \| 0.0069 \| 1.66 \| 0.7616 \| 0.93 \| 0.86 \| \| KLF2 \| 0.0060 \| 0.0089 \| 1.68 \| 0.6334 \| 0.86 \| 0.92 \| \| CXCL13 \| 0.0106 \| 0.0155 \| 0.36 \| 0.4326 \| 0.74 \| 1.67 \| \| IL2RA \| 0.0135 \| 0.0193 \| 1.55 \| 0.0871 \| 0.29 \| 2.76 \| \| ITGA4 \| 0.0133 \| 0.0193 \| 1.38 \| 0.5728 \| 0.81 \| 1.34 \| \| CD80 \| 0.0216 \| 0.0304 \| 0.42 \| 0.1483 \| 0.36 \| 1.84 \| \| IL23A \| 0.0220 \| 0.0306 \| 2.50 \| 0.6928 \| 0.87 \| 2.00 \| \| TNFSF12 \| 0.0262 \| 0.0359 \| 1.48 \| 0.3462 \| 0.63 \| 0.91 \| \| LILRA3 \| 0.0276 \| 0.0374 \| 1.57 \| 0.8285 \| 0.95 \| 1.18 \| \| LILRA1 \| 0.0280 \| 0.0375 \| 0.31 \| 0.4646 \| 0.75 \| 0.62 \| \| CCL23 \| 0.0344 \| 0.0455 \| 0.42 \| 0.3250 \| 0.62 \| 1.50 \| \| CD40LG \| 0.0396 \| 0.0517 \| 1.48 \| 0.6964 \| 0.87 \| 1.26 \| \| ADA \| 0.0437 \| 0.0557 \| 1.30 \| 0.9885 \| 1.00 \| 0.93 \| \| IDO1 \| 0.0437 \| 0.0557 \| 1.34 \| 0.1118 \| 0.33 \| 1.73 \| \| AHR \| 0.0482 \| 0.0607 \| 1.28 \| 0.5151 \| 0.78 \| 1.17 \| \| DUSP4 \| 0.0505 \| 0.0628 \| 3.28 \| 0.0299 \| 0.29 \| 3.63 \| \| PDCD1LG2 \| 0.1326 \| 0.1630 \| 2.27 \| 0.5709 \| 0.81 \| 1.59 \| \| CCL22 \| 0.1470 \| 0.1729 \| 0.94 \| 0.6541 \| 0.87 \| 1.44 \| \| CD1D \| 0.1462 \| 0.1729 \| 0.41 \| 0.6253 \| 0.86 \| 0.76 \| \| IL10 \| 0.1470 \| 0.1729 \| 1.76 \| 0.5193 \| 0.78 \| 1.23 \| \| SIGIRR \| 0.1426 \| 0.1729 \| 0.88 \| 0.4604 \| 0.75 \| 0.87 \| \| HLA.DMB \| 0.1615 \| 0.1878 \| 1.23 \| 0.7837 \| 0.93 \| 1.15 \| \| CCL2 \| 0.1660 \| 0.1889 \| 0.65 \| 0.4965 \| 0.78 \| 1.59 \| \| PYCARD \| 0.1643 \| 0.1889 \| 0.87 \| 0.8491 \| 0.97 \| 0.86 \| \| ALDH1A1 \| 0.1770 \| 0.1993 \| 1.51 \| 0.8951 \| 0.97 \| 0.95 \| \| HLA.DMA \| 0.2209 \| 0.2462 \| 1.35 \| 0.8738 \| 0.97 \| 0.94 \| \| CCR2 \| 0.2419 \| 0.2668 \| 0.46 \| 0.2060 \| 0.45 \| 0.81 \| \| SELPLG \| 0.3421 \| 0.3735 \| 0.60 \| 0.3570 \| 0.64 \| 0.66 \| \| TNFRSF9 \| 0.4347 \| 0.4698 \| 1.29 \| 0.1360 \| 0.35 \| 2.14 \| \| LAMP3 \| 0.4412 \| 0.4721 \| 0.44 \| 0.0930 \| 0.29 \| 2.19 \| \| BATF3 \| 0.4652 \| 0.4928 \| 1.06 \| 0.4384 \| 0.74 \| 1.59 \| \| CISH \| 0.4988 \| 0.5233 \| 1.48 \| 0.1543 \| 0.36 \| 2.55 \| \| EBI3 \| 0.6138 \| 0.6376 \| 1.38 \| 0.3287 \| 0.62 \| 1.83 \| \| MSR1 \| 0.6240 \| 0.6420 \| 0.87 \| 0.4733 \| 0.76 \| 1.50 \| \| GAS6 \| 0.6462 \| 0.6585 \| 1.02 \| 0.9509 \| 0.98 \| 0.97 \| \| TNFRSF4 \| 0.9430 \| 0.9519 \| 1.03 \| 0.1558 \| 0.36 \| 1.93 \| \| CYB561 \| 0.9601 \| 0.9601 \| 0.82 \| 0.6823 \| 0.87 \| 1.10 \| |
| --- | --- | --- | --- | --- | --- | --- | --- | --- | --- | --- | --- | --- | --- | --- | --- | --- | --- | --- | --- | --- | --- | --- | --- | --- | --- | --- | --- | --- | --- | --- | --- | --- | --- | --- | --- | --- | --- | --- | --- | --- | --- | --- | --- | --- | --- | --- | --- | --- | --- | --- | --- | --- | --- | --- | --- | --- | --- | --- | --- | --- | --- | --- | --- | --- | --- | --- | --- | --- | --- | --- | --- | --- | --- | --- | --- | --- | --- | --- | --- | --- | --- | --- | --- | --- | --- | --- | --- | --- | --- | --- | --- | --- | --- | --- | --- | --- | --- | --- | --- | --- | --- | --- | --- | --- | --- | --- | --- | --- | --- | --- | --- | --- | --- | --- | --- | --- | --- | --- | --- | --- | --- | --- | --- | --- | --- | --- | --- | --- | --- | --- | --- | --- | --- | --- | --- | --- | --- | --- | --- | --- | --- | --- | --- | --- | --- | --- | --- | --- | --- | --- | --- | --- | --- | --- | --- | --- | --- | --- | --- | --- | --- | --- | --- | --- | --- | --- | --- | --- | --- | --- | --- | --- | --- | --- | --- | --- | --- | --- | --- | --- | --- | --- | --- | --- | --- | --- | --- | --- | --- | --- | --- | --- | --- | --- | --- | --- | --- | --- | --- | --- | --- | --- | --- | --- | --- | --- | --- | --- | --- | --- | --- | --- | --- | --- | --- | --- | --- | --- | --- | --- | --- | --- | --- | --- | --- | --- | --- | --- | --- | --- | --- | --- | --- | --- | --- | --- | --- | --- | --- | --- | --- | --- | --- | --- | --- | --- | --- | --- | --- | --- | --- | --- | --- | --- | --- | --- | --- | --- | --- | --- | --- | --- | --- | --- | --- | --- | --- | --- | --- | --- | --- | --- | --- | --- | --- | --- | --- | --- | --- | --- | --- | --- | --- | --- | --- | --- | --- | --- | --- | --- | --- | --- | --- | --- | --- | --- | --- | --- | --- | --- | --- | --- | --- | --- | --- | --- | --- | --- | --- | --- | --- | --- | --- | --- | --- | --- | --- | --- | --- | --- | --- | --- | --- | --- | --- | --- | --- | --- | --- | --- | --- | --- | --- | --- | --- | --- | --- | --- | --- | --- | --- | --- | --- | --- | --- | --- | --- | --- | --- | --- | --- | --- | --- | --- | --- | --- | --- | --- | --- | --- | --- | --- | --- | --- | --- | --- | --- | --- | --- | --- | --- | --- | --- | --- | --- | --- | --- | --- | --- | --- | --- | --- | --- | --- | --- | --- | --- | --- | --- | --- | --- | --- | --- | --- | --- | --- | --- | --- | --- | --- | --- | --- | --- | --- | --- | --- | --- | --- | --- | --- | --- | --- | --- | --- | --- | --- | --- | --- | --- | --- | --- | --- | --- | --- | --- | --- | --- | --- | --- | --- | --- | --- | --- | --- | --- | --- | --- | --- | --- | --- | --- | --- | --- | --- | --- | --- | --- | --- | --- | --- | --- | --- | --- | --- | --- | --- | --- | --- | --- | --- | --- | --- | --- | --- | --- | --- | --- | --- | --- | --- | --- | --- | --- | --- | --- | --- | --- | --- | --- | --- | --- | --- | --- | --- | --- | --- | --- | --- | --- | --- | --- | --- | --- | --- | --- | --- | --- | --- | --- | --- | --- | --- | --- | --- | --- | --- | --- | --- | --- | --- | --- | --- | --- | --- | --- | --- | --- | --- | --- | --- | --- | --- | --- | --- | --- | --- | --- | --- | --- | --- | --- | --- | --- | --- | --- | --- | --- | --- | --- | --- | --- | --- | --- | --- | --- | --- | --- | --- | --- | --- | --- | --- | --- | --- | --- | --- | --- | --- | --- | --- | --- | --- | --- | --- | --- | --- | --- | --- | --- | --- | --- | --- | --- | --- | --- | --- | --- | --- | --- | --- | --- | --- | --- | --- | --- | --- | --- | --- | --- | --- | --- | --- | --- | --- | --- | --- | --- | --- | --- | --- | --- | --- | --- | --- | --- | --- | --- | --- | --- | --- | --- | --- | --- | --- | --- | --- | --- | --- | --- | --- | --- | --- | --- | --- | --- | --- | --- | --- | --- | --- | --- | --- | --- | --- | --- | --- | --- | --- | --- | --- | --- | --- | --- | --- | --- | --- | --- | --- | --- | --- | --- | --- | --- | --- | --- | --- | --- | --- | --- | --- | --- | --- | --- | --- | --- | --- | --- | --- | --- | --- | --- | --- | --- | --- | --- | --- | --- | --- | --- | --- | --- | --- | --- | --- | --- | --- | --- | --- | --- | --- | --- | --- | --- | --- | --- | --- | --- | --- | --- | --- | --- | --- | --- | --- | --- | --- | --- | --- | --- | --- | --- | --- | --- | --- | --- | --- | --- | --- | --- | --- | --- | --- | --- | --- | --- | --- | --- | --- | --- | --- | --- | --- | --- | --- | --- | --- | --- | --- | --- | --- | --- | --- | --- | --- | --- | --- | --- | --- | --- | --- | --- | --- | --- | --- | --- | --- | --- | --- | --- | --- | --- | --- | --- |

**Supplementary Table S8**. List of differentially expressed genes between LTBI and TB that are induced by IL-1b, with their respective p and q values, as well as fold-change.
