# Supplementary figures and images for: Tuberculosis alters immune-metabolic pathways resulting in perturbed IL-1 responses
Source: Front Immunol. 2022 Dec 14;13:897193. doi: 10.3389/fimmu.2022.897193 (PMC9795069; doi:10.3389/fimmu.2022.897193)

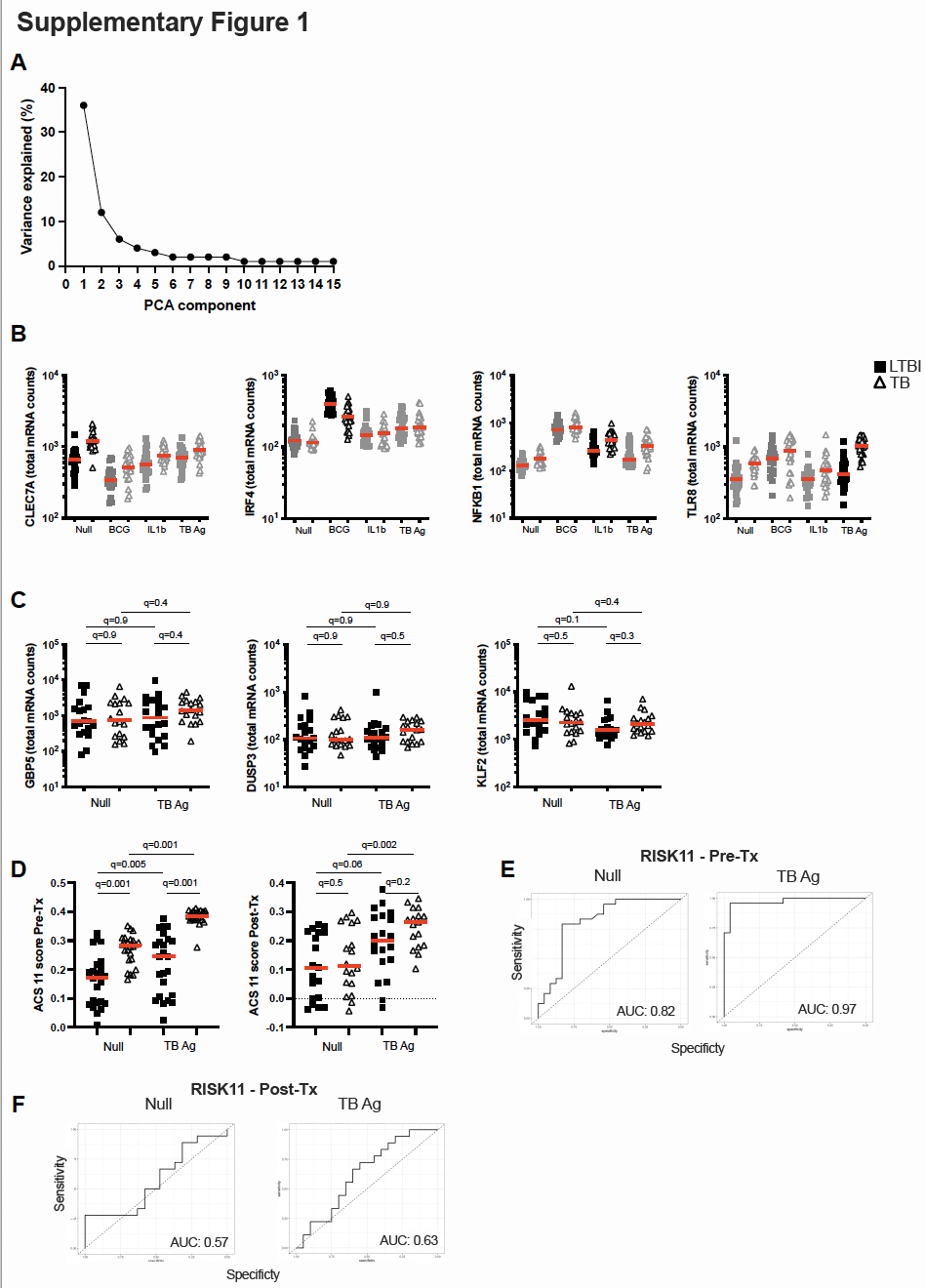

Supplement: Supplementary Figure 1 — (A) Scree plot showing the variance explained by each PCA component on the gene expression (Nanostring) dataset. (B) Examples of significantly different (q<0.001) stimuli-specific induced transcripts between TB and LTBI. (C) Expression of the 3 genes from Sweeney3 (69) in the Null and TB Ag conditions on visit 2 post-treatment. (D) RISK11 score (18) in the Null and TB Ag conditions, pre and post-treatment, for the LTBI and TB groups, calculated using the Singscore method. Area under the receiver operating characteristic curve (AUC) for discrimination of LTBI/TB groups using the RISK11 score for the Null and TB Ag conditions on visit 1 pre-treatment (Pre-Tx) (E) and visit 2 after successful antibiotic treatment (Post-Tx) (F). Comparisons of LTBI/TB groups within the same stimulation were performed using unpaired non-parametric t-tests; comparisons between Null and stimulated conditions within the LTBI/TB groups were performed using a paired non-parametric t-test. Correction for multiple comparisons was then applied. Red line: median values. Closed square: LTBI, Open triangle: TB. [file Image_1.jpeg]

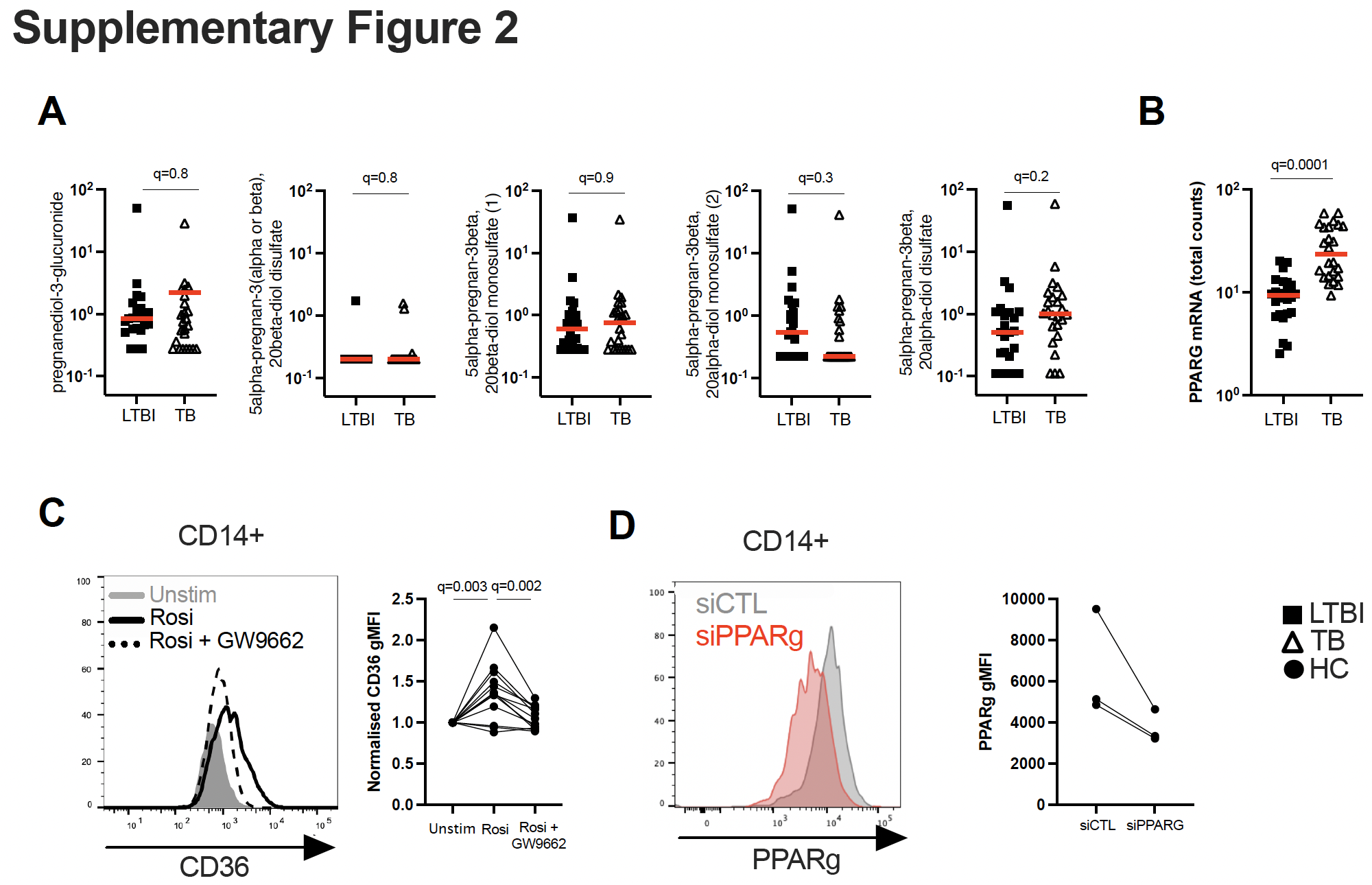

Supplement: Supplementary Figure 2 — (A) Relative levels of pregnane steroids identified in for both LTBI and TB, measured by mass spectrometry in the Null condition. (B) Gene expression levels of PPARG in the Null condition measured by Nanostring both in LTBI and TB. (C) Protein levels of CD36 measured by flow cytometry on the surface of CD14+ monocytes from healthy individuals in the presence or absence of the PPARγ agonist rosiglitazone (Rosi) and/or the PPARγ antagonist GW9662 (n=13, 5 independent experiments). (D) Intracellular protein levels of PPARγ measured by flow cytometry in CD14+ monocytes from healthy individuals after treatment with PPARG small interfering RNA (siRNA) or control (n=3, 2 independent experiments). Comparisons between groups were performed using unpaired (A, B) or paired (C) non-parametric t-tests and correction for multiple comparisons was applied. Red line: median values. Closed square: LTBI, Open triangle: TB, Circle: Healthy Control (HC). [file Image_2.jpeg]
